# Artificial neural network based hierarchical intelligent control framework for a residential microgrid

**DOI:** 10.1038/s41598-025-29034-x

**Published:** 2025-11-23

**Authors:** Mohammed O. Bahabri, Sreerama Kumar Ramdas, Hussam A. Banawi

**Affiliations:** Department of Electrical and Computer Engineering, Faculty of Engineering, King Abdulaziz University, Jeddah, Saudi Arabia

**Keywords:** Neural network control, Hybrid microgrid, Energy management system, MATLAB/Simulink, Saudi Arabia, Energy science and technology, Engineering

## Abstract

As Saudi Arabia accelerates its transition to intelligent, sustainable energy systems under Vision 2030, advanced control of renewable microgrids becomes critical. This study proposes an artificial neural network-based hierarchical intelligent control framework for a fully renewable hybrid microgrid powering a residential villa in Jeddah, Saudi Arabia. The system integrates a 36 kW solar PV array, 10 kW wind turbine system, and 100 kWh lithium-ion battery to meet a daily load of 177.5 kWh. Neural networks are embedded across all control layers: MLP-based MPPT for solar and wind sources, NARMA-L2 for battery management, and an intelligent EMS for system-wide coordination. Simulations conducted in MATLAB/Simulink, under realistic variable irradiance (0.1–0.8 kW/m²) and wind speeds (3.5–5 m/s) characteristic of Jeddah, quantitatively demonstrate the superior performance of the proposed framework. Specifically, the MLP-based MPPT controllers achieves a power tracking enhancement of up to 12% compared to conventional MPPT methods, significantly improving energy harvesting efficiency. The NARMA-L2 battery management controller exhibits a reduction in DC bus voltage error by approximately 10% and an improvement in recovery time by over 5% when benchmarked against traditional PI control, underscoring its enhanced stability and responsiveness. Furthermore, the intelligent EMS successfully maintained real-time load balancing and optimized battery operation across five distinct dynamic load scenarios, confirming its robustness. The simulation results validate the effectiveness, resilience, and scalability of the proposed neural network-based hierarchical control system, positioning it as a promising solution for smart residential microgrids in challenging coastal environments.

**Supplementary Information:**

The online version contains supplementary material available at 10.1038/s41598-025-29034-x.

##  Introduction

Saudi Arabia is undergoing a significant energy transition, driven by the national goals of economic diversification and sustainability outlined in Vision 2030^1^. This transition emphasizes the adoption of renewable energy and artificial intelligence (AI) as key enablers of a modern, intelligent energy infrastructure^2,3^. However, the increasing residential energy demand, particularly in coastal cities like Jeddah, presents a substantial challenge to grid stability due to high air-conditioning loads and rapid urbanization^4^. While hybrid microgrids integrating solar photovoltaic (PV), wind turbines, and battery energy storage systems (BESS) offer a viable solution for enhancing energy resilience, their effectiveness is often constrained by the inherent intermittency of renewable sources and the complexities of achieving real-time power balancing and control^5^. Conventional control methods struggle to adapt to the dynamic and nonlinear nature of these systems, leading to suboptimal performance and potential instability. This study addresses this critical gap by proposing a neural network-based hierarchical control framework designed to enhance the adaptability, voltage stability, and energy optimization of a fully renewable residential microgrid in Jeddah. Our central research question is: How can a multi-layered neural network architecture be effectively integrated across primary, secondary, and tertiary control levels to achieve robust, autonomous, and optimized operation of a residential microgrid under the variable environmental and load conditions characteristic of Saudi Arabia’s coastal regions?

A range of studies have investigated the design, control, and optimization of microgrids in the context of Saudi Arabia, with varying degrees of integration of renewable energy sources and artificial intelligence (AI). This review critically evaluates relevant publications, examining their technical approaches, application domains, and contributions to advancing intelligent, sustainable energy systems in the region.

The application of artificial neural networks (ANNs) in fully renewable DC and AC microgrids features prominently in several studies. For example^6^, presents an ANN-based nonlinear control strategy for a PV–battery DC microgrid, significantly enhancing system response under nonlinear operating conditions. Building on this^7^, proposes a hybrid control method combining ANN, proportional–integral (PI), and droop control, which improves voltage regulation and dynamic adaptability in AC systems. Similarly^8^, employs a neural-fuzzy EMS architecture that integrates fuzzy logic with particle swarm optimization, achieving improved energy efficiency and operational cost reductions in AI-controlled systems.

AI-enabled control of hybrid storage architectures has also gained attention^9^. introduces an EMS for a hydrogen-based microgrid integrating PV, batteries, and fuel cells, where AI-based coordination facilitates predictive balancing and enhances overall reliability. Addressing communication latency challenges in DC systems^10^, applies a backward neural network–driven multilevel control scheme to maintain voltage stability. Meanwhile^11^, combines deep learning with sliding mode control in a robust control framework tailored to DC microgrids with renewable energy.

In terms of AI-optimized controllers^12^, develops an adaptive PID controller trained via the Honey Badger algorithm to support frequency regulation under dynamic load conditions in fully renewable microgrids. Likewise^13^, presents a nonlinear ANN-based EMS for PV-fed DC systems that enhances maximum power point tracking (MPPT) and load management during solar variability. This trend extends to residential applications in^14^, where a neural network–based EMS optimizes energy dispatch and supply–demand matching in real time.

While many of the reviewed systems are AI-powered, a subset of studies contribute by focusing on optimization without machine learning techniques. For instance^15^, analyses a real-world grid-connected microgrid in Saudi Arabia designed to meet the electricity demands of an industrial building in Saudi Arabia. Similarly^16^, introduces a techno-economic optimization approach for sizing hybrid PV–wind–battery systems for residential use, offering a feasible design despite the absence of AI.

A shift toward advanced AI in energy management becomes apparent in^17^, which applies EMS techniques based on cutting edge fuzzy-based smart energy management system adapted to Saudi residential buildings. Reinforcement learning is explored in^18^, where a hybrid system for a remote community integrates solar, wind, and batteries, achieving high renewable penetration despite using conventional control. Broader implications are discussed in^19^, a national review of AI in Saudi smart grid development, highlighting the importance of intelligent control systems for achieving Vision 2030 targets. Complementing this^20^, assesses infrastructure challenges and regulatory gaps affecting renewable integration, offering valuable insights for future microgrid deployment.

Forecasting remains a critical application of AI, especially for operational planning. In^21^, a study focusing on Yanbu City demonstrated that an optimally designed hybrid microgrid comprising solar photovoltaic (PV), wind turbines, battery storage, and grid connectivity can achieve over 82% renewable energy penetration in meeting the city’s energy demands, though it lacks AI-based forecasting or control. To address such limitations^22^, applies ANN and support vector regression (SVR) models for short-term load prediction, showing clear gains in accuracy and planning efficiency. On a broader scale^23^, employs GIS-based tools to assess national renewable potential for PV and wind microgrids, aiding spatial planning efforts.

Residential energy management with adaptive AI control is exemplified in^24^, where an EMS improves self-consumption and flexibility in a smart microgrid. In contrast^16^, targets off-grid communities in remote desert areas, using a techno-economic model without AI but successfully demonstrating energy access potential and hybrid system viability.

Microgrid stability and power quality are also enhanced through AI-based techniques^25^. applies ANN controllers to improve voltage regulation and dynamic performance in renewable microgrids. A simulation study in^26^ confirms the viability of a PV–battery system in a Riyadh residential setting, highlighting both technical and financial suitability for national implementation goals. Regulatory and operational guidelines are explored in^27^, which emphasizes enabling policies and infrastructure planning for microgrid deployment in Saudi Arabia.

Emerging research on predictive maintenance leverages AI to further improve system resilience^28^. develops models capable of identifying early-stage faults in PV and wind components, thereby extending component lifetimes and reducing unplanned downtime. Finally^29^, presents a comprehensive economic feasibility analysis for microgrids across Saudi urban and rural regions, reinforcing the scalability of renewable-based systems for both centralized and decentralized applications.

The reviewed literature clearly shows the progress made in deploying renewable energy-based microgrids in Saudi Arabia, with increasing interest in applying artificial intelligence to enhance control, forecasting, and energy management. Numerous studies have investigated renewable energy configurations using solar, wind, hydrogen, and storage systems, while others have introduced AI techniques such as artificial neural networks, machine learning, and deep reinforcement learning. However, these efforts often remain limited in scope, either applying AI to isolated control functions such as MPPT, load forecasting, or energy scheduling or focusing solely on system design without incorporating intelligent control strategies.

Despite the growing interest in AI-driven microgrid solutions, a significant gap persists in the literature concerning the comprehensive and hierarchical integration of neural network-based control across all operational layers—primary, secondary, and tertiary—within the specific context of Saudi Arabia. Existing studies often focus on isolated AI applications (e.g., MPPT, load forecasting) or system design without a unified, intelligent control architecture. This work distinguishes itself by presenting a novel, fully integrated neural network-based hierarchical control framework for a residential microgrid in Jeddah, Saudi Arabia. Unlike previous approaches, our framework embeds distinct neural network models at each control level: Multi-Layer Perceptron (MLP) for Maximum Power Point Tracking (MPPT) in solar PV and wind systems, Nonlinear Autoregressive Moving Average with Exogenous Inputs (NARMA-L2) for advanced battery management, and an intelligent Energy Management System (EMS) for system-wide coordination. This multi-layered neural network collaborative control approach represents a significant methodological innovation, moving beyond conventional control strategies and fragmented AI applications. The proposed microgrid controller aims to minimize three conflicting objectives: DC-bus voltage deviation, operating cost, and battery state-of-charge (SOC) constraint violations. These objectives are subject to the power-balance constraint (generation + battery discharge = load + battery charge), converter current/voltage limits, and SOC bounds. Maintaining voltage stability under fluctuating renewable generation and load is challenging poor tuning can lead to increased settling times and oscillations. Therefore, a hierarchical control structure is designed in which primary controllers (MPPT) maximise generation, a secondary controller (neuro-NARMA-L2) regulates DC-bus voltage, and a tertiary neural-network-based Energy Management System (EMS) schedules battery usage to reduce costs. The integrated neural network approach adapts to fast dynamics at the converter level and slower economic considerations at the EMS level, providing a unified solution for voltage regulation, cost minimisation and battery health.

 The rest of this paper is organized as follows: Sect. 2 presents the rationale for selecting the study location Jeddah, Saudi Arabia including an overview of the local solar irradiation and wind profiles. It also outlines the optimal sizing of the microgrid components based on local resource availability and residential load requirements. Section 3 describes the system modelling and introduces the neural network-based hierarchical control architecture designed to manage the integration and coordination of distributed energy resources. Section 4 describes a fictitious day scenario, outlining the dynamic interactions between electricity production and household consumption in varying environmental conditions. Section 5 concludes the manuscript with an overall summary of the aspects of principal findings and the suggestion of future research effort in the direction to employ scale and real-world deployment.

## System study and optimal sizing

**Fig. 1:**
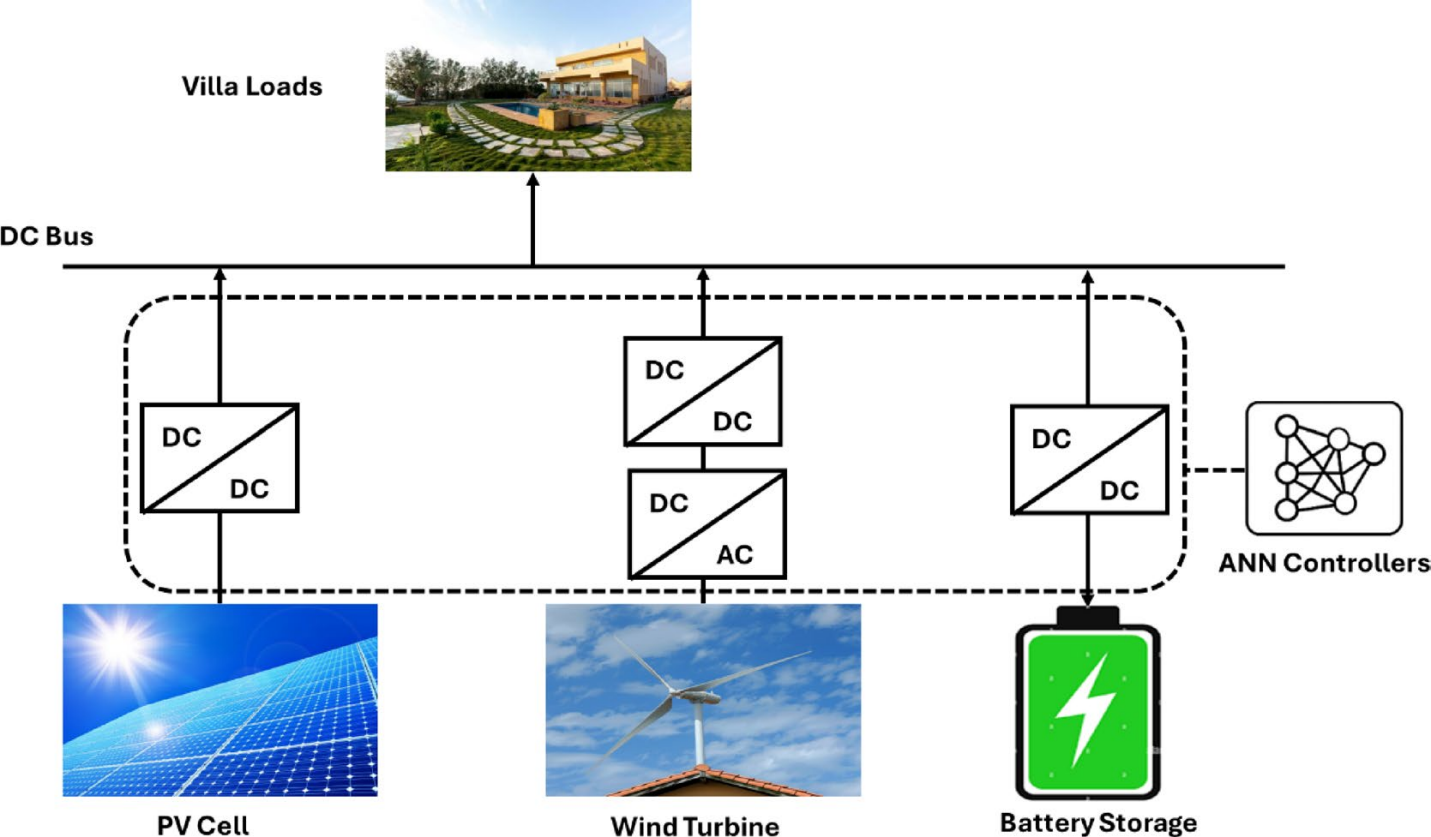
Architecture of the Microgrid.

The current section defines the study site, the estimation of residential energy demand and optimal sizing of a stand-alone renewable energy system submitted by photovoltaic (PV) panels, wind turbines and battery storage, as shown in Fig. 1.

Jeddah benefits from lots of sun. It has sun for 5.8 to 6.2 kWh/m²/day. Additionally, it gets windy by the sea. The wind speed is 4 to 5.5 m/s, especially at night. This wind speed makes it advantageous to use both solar and wind power. The load scenario refers to the power demand of the house, which includes a significant amount of air conditioning and various technological devices. It’s got a pool and smart stuff. It charges an electric car too. We obtained a list of all electrical items from one of the citizens in that region as presented in Table 1. They checked how much power each item needs. They checked how long each is used per day. They worked out how much power is all used in a day. It adds up to about 177.5 kWh. The most power is used at night.

**Table 1:** Estimated daily energy Consumption.

Appliance/equipment	Power rating (W)	Daily use (hrs)	Daily energy (kWh)
Air Conditioning (4 Split + 1 Central Unit)	9500	12	114.0
Refrigerator (Double Door + Mini Bar)	400	24	9.6
Lighting (Indoor & Outdoor)	800	10	8.0
Washing Machine	600	1	0.6
Dishwasher	1500	1	1.5
Electric Oven	3000	1	3.0
Microwave	1200	0.5	0.6
Televisions (4 Units)	600	6	3.6
Computers/Workstations (3 Units)	600	8	4.8
Water Heaters (2 Instant + 1 Tank)	7000	2	14.0
Vacuum Cleaner	1200	0.5	0.6
Kitchen Appliances	800	1	0.8
Outdoor Lighting & Smart Garden	600	10	6.0
Electric Vehicle Charger (7.4 kW)	7400	2	14.8
Pool Pump and Filtration	1200	6	7.2
Wi-Fi & Smart Systems	200	24	4.8
Total Estimated Daily Consumption	—	—	177.5 kWh

In order to meet the estimated daily energy demand of the villa, which is 177.5 kWh, the proposed microgrid includes PV panels, wind turbines and a lithium ion battery energy storage system (BESS). The procedure follows a HOMER backbone of similar energy balance with conversion of local renewable resource information, design safety factors and autonomy requirements to achieve reliable off grid operation.

The PV array is designed to supply 70% of the daily load, corresponding to 124.25 kWh/day. Given Jeddah’s average solar irradiance of 5.8 kWh/m²/day and a system efficiency of 75%, the nominal PV capacity is calculated as:

1 $$P_{{PV}} = \frac{{~124.25}}{{\left( {5.8 \times 0.75} \right)}} = 28.5\;{\mathrm{kW}}$$

To ensure robustness against seasonal variation, an oversizing factor of 1.25 is applied:

2 $$P_{{PVfinal}} = 1.25 \times 28.5 = 35.6\;{\mathrm{kW}}$$

This results in a final PV capacity of 36 kW, achievable using approximately 124 modules of 300 W each. This array is expected to serve as the primary generation unit due to the high solar potential in the region.

Wind energy supplements solar generation with a target contribution of 20% (35.5 kWh/day). Assuming a 5 kW turbine with a 20% capacity factor, the daily energy production is estimated as:

3 $$E_{{wind}} = 5 \times 24 \times 0.20 = 24\;{\mathrm{kWh}}/{\mathrm{day}}$$

Deploying two 5 kW turbines (totalling 10 kW) results in a total wind energy contribution of 48 kWh/day. This exceeds the target and introduces redundancy into the system while helping cover night-time and low-irradiance conditions.

The BESS is designed to provide 10 h of energy autonomy. Given an average load of 7.4 kW, the required storage capacity is:

4 $$E_{{BESS}} = 10 \times 7.4 = 74\;{\mathrm{kWh}}$$

With a depth of discharge (DoD) of 80% and a buffer factor of 1.2 for aging and uncertainties, the total battery capacity required is:

5 $$C_{{battery}} = \frac{{74}}{{0.8}} \times 1.2 = 111\;{\mathrm{kWh}}$$

This is rounded to a commercial capacity of 100 kWh, achievable through modular lithium-ion battery systems. This storage capacity ensures system reliability during peak demand or periods of low generation. The Table 2 below summarize the sizing calculations for the microgrid system.

**Table 2:** Summary of microgrid component Sizing.

Component	Target contribution	Daily energy target (kWh)	Final capacity
Photovoltaic (PV)	70% of 177.5 kWh	124.25	36 kW
Wind Turbines	20% of 177.5 kWh	35.5	10 kW
Battery Storage	10 h autonomy	73.96	100 kWh

## System modelling and control architecture

In this work, the studied microgrid comprises a Photovoltaic system, a backup system composed of a battery energy storage system and electrical grid, power converters, and electrical loads. This section will present the modelling of each microgrid component and the description of each controller.

### System modelling

#### Photovoltaic system

**Fig. 2:**
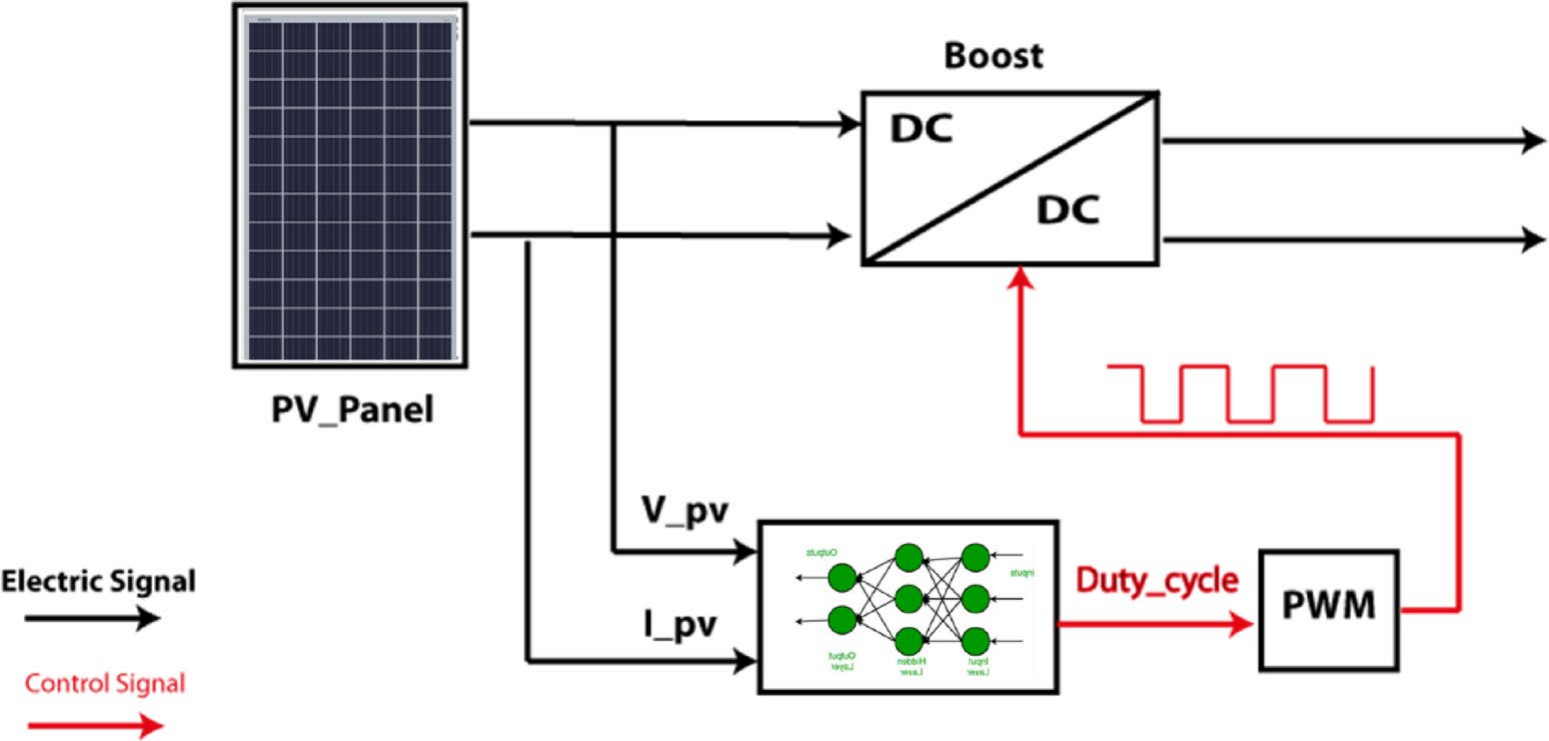
PV Cell system.

The photovoltaic system consists of a photovoltaic panel, boost converter, and a Maximum Power Point Tracking (MPPT) controller, as illustrated in Fig. 2.

**Fig. 3:**
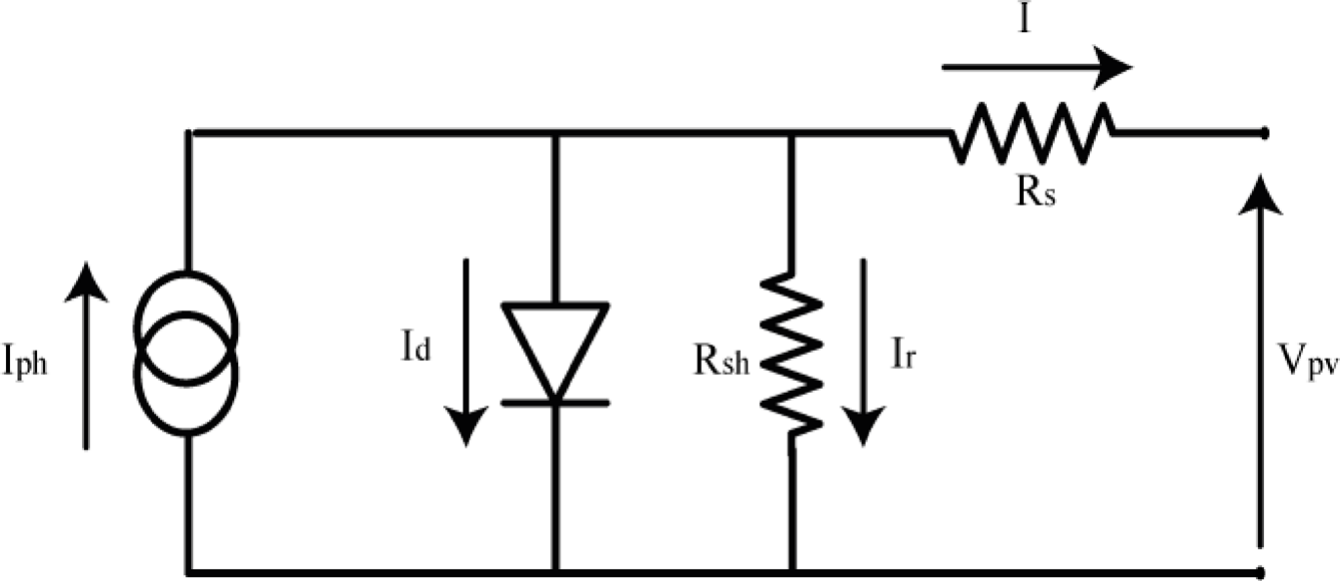
Equivalent circuit of the solar cell with a single diode.

The photovoltaic cell is an electronic component that makes it possible to produce electricity from exposure to the sun. The cell can be used alone or assembled with other solar cells to form a photovoltaic panel. Several PV cell models exist and vary in complexity and precision. The photovoltaic cell model used in this study is based on the dynamic PV model developed as shown in Fig. 3.

The following equation model the current of the PV cell:

6 $$I_{{pv}} = I_{{ph}} - I_{0} \left[ {\exp \left( {\frac{{q{\mathrm{~}}V_{d} }}{{K_{b} {\mathrm{~}}F{\mathrm{~}}T_{c} }}} \right) - 1} \right] - \frac{{V_{d} }}{{R_{p} }}$$

Iph is the light generated current or photocurrent, Io is the cell saturation current dependent on the cell temperature, q is the electric charge (1.6 × 10–19 C), Kb is the Boltzmann’s constant, F is the cell idealizing factor, Tc is the cell’s absolute temperature, Vd is the diode voltage, and Rp is the parallel resistance. In this work, we used an implemented PV model at MATLAB/Simulink (Sun Power SPR-250NX-BLK-D) as a reference module.

Photovoltaic arrays have limited conversion efficiency. For this reason, a maximum power point tracking technique is essential. To extract the maximum output power from PV modules, MPPT techniques are utilized, and different DC/DC converter topologies are used to transfer the maximum power from PV modules to loads.

#### Wind turbine system

Wind energy, after solar energy, is one of the most abundant and promising renewable energy resources available for electricity generation Wind energy is very abundant, particularly in coastal areas like Jeddah, making it a useful part of hybrid renewable energy systems. Wind turbines use the kinetic energy generated by the airstream and it is converted into mechanical energy using rotor blades, which are connected to the shaft. The resulting mechanical energy is then converted to electrical energy by a generator, which is normally a synchronous permanent magneto-generator (PMSG), due to its high efficiency, reliability and negligible maintenance requirements.

Wind speed is a major driving factor for the turbine, with the turbine power increasing in proportion to the wind speed cubed up to the rated wind speed. In order to get the most energy out of the wind in variable wind conditions, the turbine must be running at its maximum power point (MPP), which requires an efficient Maximum Power Point Tracking (MPPT) algorithm embedded in the power-electronic interface.

**Fig. 4:**
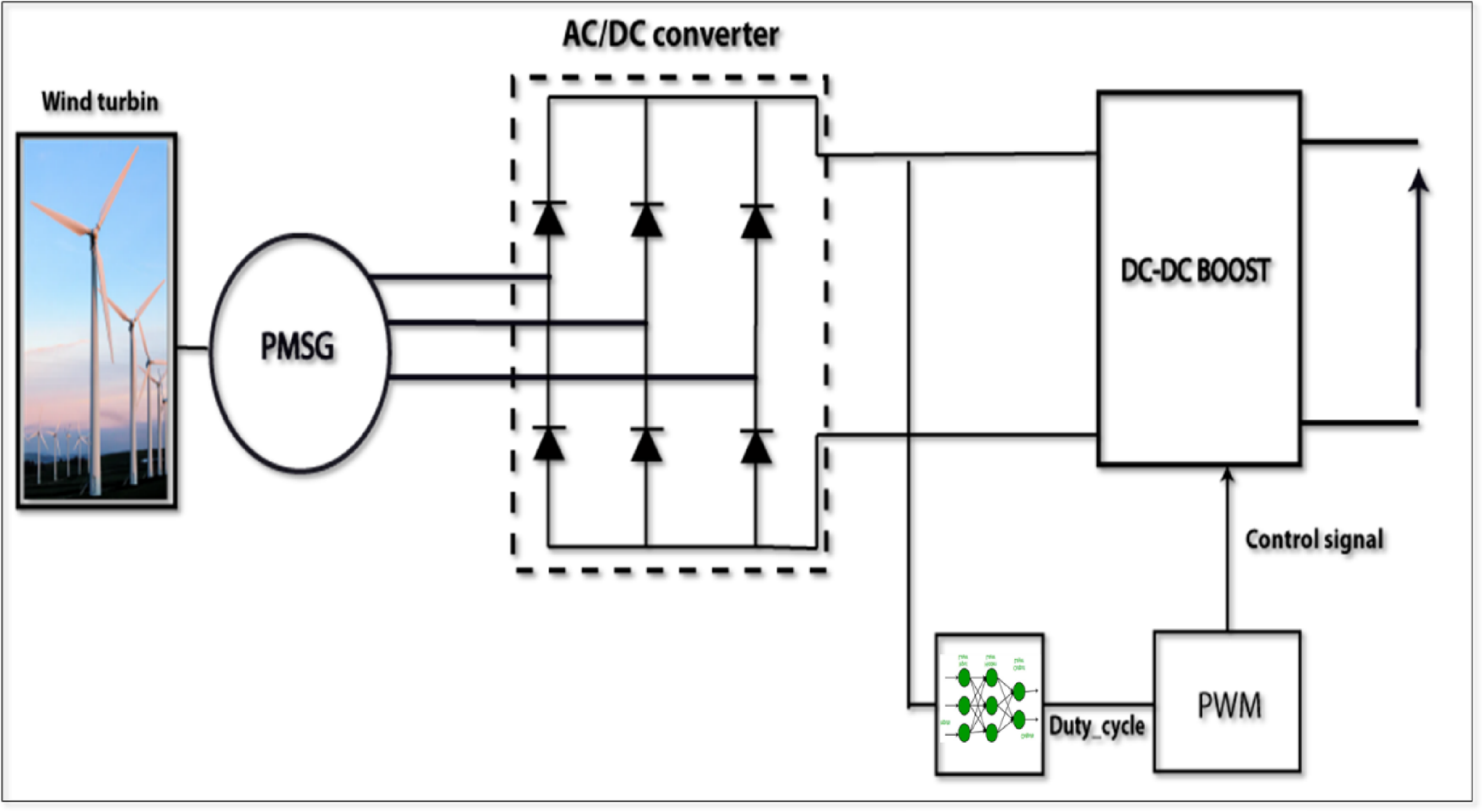
Wind power system.

As shown in Fig. 4, the wind power system in this study consists of a three-phase diode bridge rectifier that converts the variable-frequency AC voltage generated by the wind turbine into a pulsating DC voltage. This DC voltage is then processed by a DC-DC boost converter equipped with MPPT control to regulate the output and ensure operation near the MPP. The MPPT technique used is analogous to that applied in PV systems but adapted to account for wind dynamics and turbine characteristics.

The wind turbine model is interfaced with the DC bus of the microgrid and coordinated with other renewable sources and the battery storage system through the centralized control architecture. Under low load or full battery conditions, the EMS may curtail wind power to maintain system stability and avoid unnecessary stress on converters or storage elements.

#### Battery energy storage system (BESS)

**Fig. 5:**
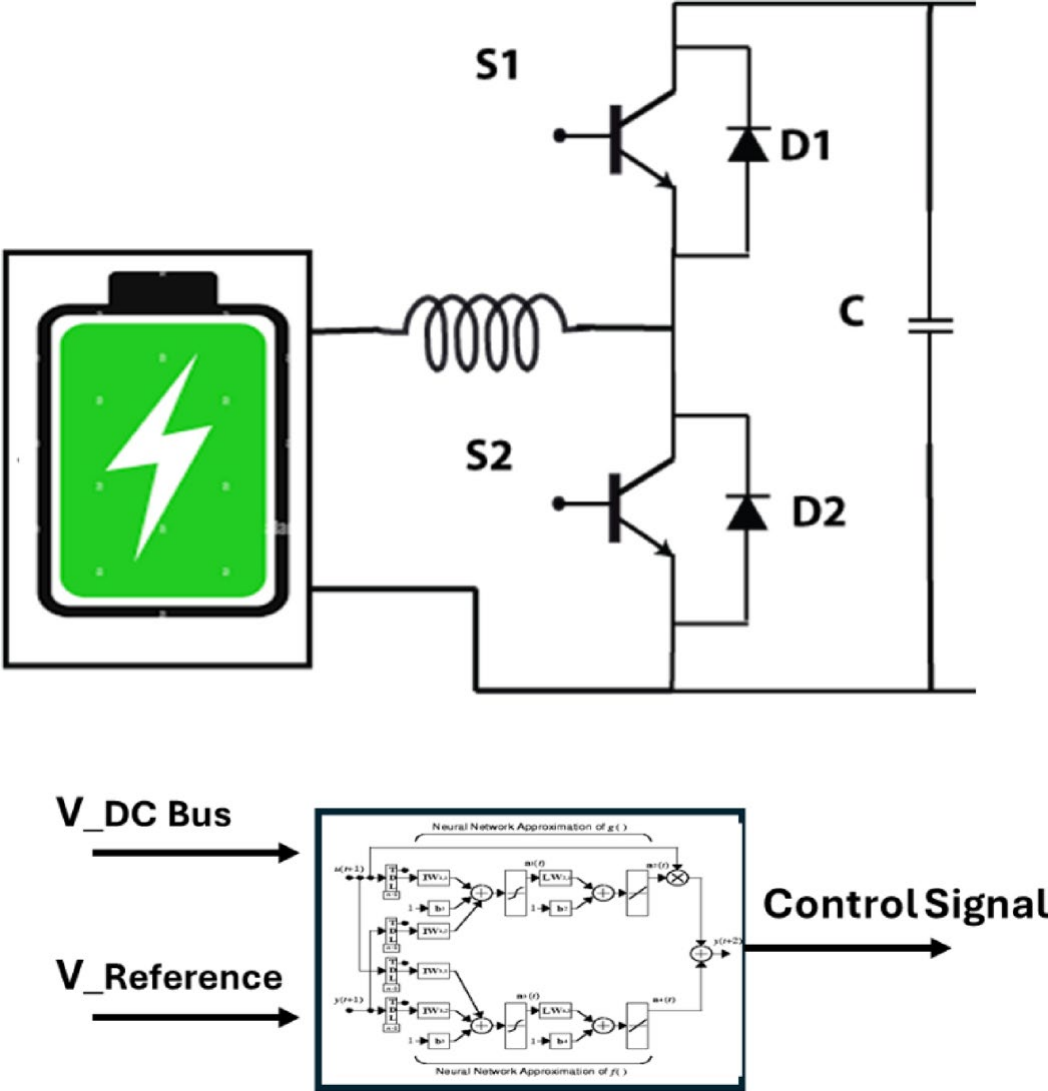
Battery energy storage system (BESS).

The Battery Energy Storage System (BESS) is used as a backup power source when the solar sources are insufficient to cover the loads demand and as a load when there is surplus power to keep the system balanced. The BESS is composed of a Li-ion battery, a bidirectional DC-DC converter, and a controller to manage the charging and discharging of the battery to keep the balance at the microgrid bus, as shown in Fig. 5. Because of the nonlinearity of the voltage response, lithium-ion batteries are a difficult system to model. In this paper, we use the dynamic battery model from Simulink’s SimPowerSystems library.

The bidirectional DC/DC converter allows power to be transferred between the battery and the microgrid in either direction. They are increasingly being used as a connection between the battery and the microgrid system to manage battery charging and discharging due to their ability to reverse the direction of current flow while keeping the voltage polarity at either end unchanged.

#### Bidirectional DC/DC converter (BDC)

The bidirectional DC-DC converter used in the present study, shown in Fig. 5, is a half-bridge inverter-gate bipolar transistor (IGBT) topology in the continuous conduction mode (CCM). The converter acts in boost mode; it behaves in buck mode at the time of storing the surplus of energy on the DC bus. In boost mode, switches S2 and diode D1 are conducting, and the current is directed to DC bus. In buck mode, switches S1 and diode D2 are conducting, and power is produced for the battery.

To increase the intelligence and adaptability of the microgrid control system, artificial neural networks (ANNs) are put in place in critical control points of the architecture. Specifically, two models of neural network controllers are implemented: Multilayer Perceptron (MLP) model, which is used for the regulation of the photovoltaic (PV) and the wind subsystems and Nonlinear Auto-Regressive Moving Average model with exogenous input (NARMA-L2), which is used for the management of the battery energy storage system (BESS). These models are chosen depending on their compatibility with different control goals and dynamic system behaviours that would help the microgrid induce efficient responses to real-time variations of loads and renewable generation.

In order to establish a certain degree of reproducibility and transparency of the system design, in this section, the electrical and control parameters of all the power converters, the DC bus and the permanent magnet synchronous generator (PMSG) are described.

*PV boost converter* To interface the photovoltaic array with the DC bus, the boost converter uses a 10 mH inductor and a 0.33 mF output capacitor. It operates at a 50 kHz switching frequency with a control sampling period of 10 µs. The equivalent series resistance of its passive components is about 0.02 Ω, and the inner current-loop bandwidth is roughly 2.5 kHz. The power stage employs a straightforward LC filter using these inductance and capacitance values to smooth the output current.

*Wind converter (PMSG rectifier with boost stage)* The power converter that rectifies and boosts the output of the permanent-magnet synchronous generator (PMSG) uses a 3 mH inductor and a 0.2 mF capacitor. Its switching frequency is lower—around 20 kHz—with a 20 µs sampling period. Typical equivalent series resistance is about 0.015 Ω, and the control bandwidth is about 1 kHz. Because the wind stage has slower dynamics than the PV stage, a simple L- or LC-filter is adequate to smooth the rectified output.

*Battery bidirectional converter* Connecting the battery to the DC bus requires a buck–boost converter with a relatively large inductance (about 6.3 mH) and a 5 mF capacitor that doubles as the DC-link capacitor. The switching frequency is usually set near 20 kHz, with a sampling period of 25 µs. Component ESR is roughly 0.02 Ω, and the control bandwidth is intentionally slower—around 500 Hz—to avoid interaction with faster PV and wind controllers. A split-inductor or L-filter design is commonly used so the converter can operate efficiently in both charge and discharge modes.

*DC bus* The overall DC-link relies on a large bulk capacitor of about 5 mF to stabilise the bus voltage during transient load and generation changes. The equivalent series resistance of this capacitor is very low, around 0.005 Ω, and a damping network may be added to suppress oscillations.

*Permanent-Magnet synchronous generator (PMSG)* The wind turbine’s generator has a rated power of 10 kW. At its nominal operating point, each phase produces about 260 V electromotive force when spinning at approximately 210 rpm. This low-speed design results in an electrical frequency of 56 Hz for a three-phase machine. The air gap between rotor and stator is around 0.1 cm, with slot openings on the stator of roughly 0.3 cm. The stator’s internal resistance is about 0.27 Ω, and the machine’s efficiency at nominal conditions is roughly 92%. These parameters collectively determine the generator’s electromechanical performance and ensure it is well-matched to the DC-bus voltage when rectified.

### Hierarchical control system

**Fig. 6:**
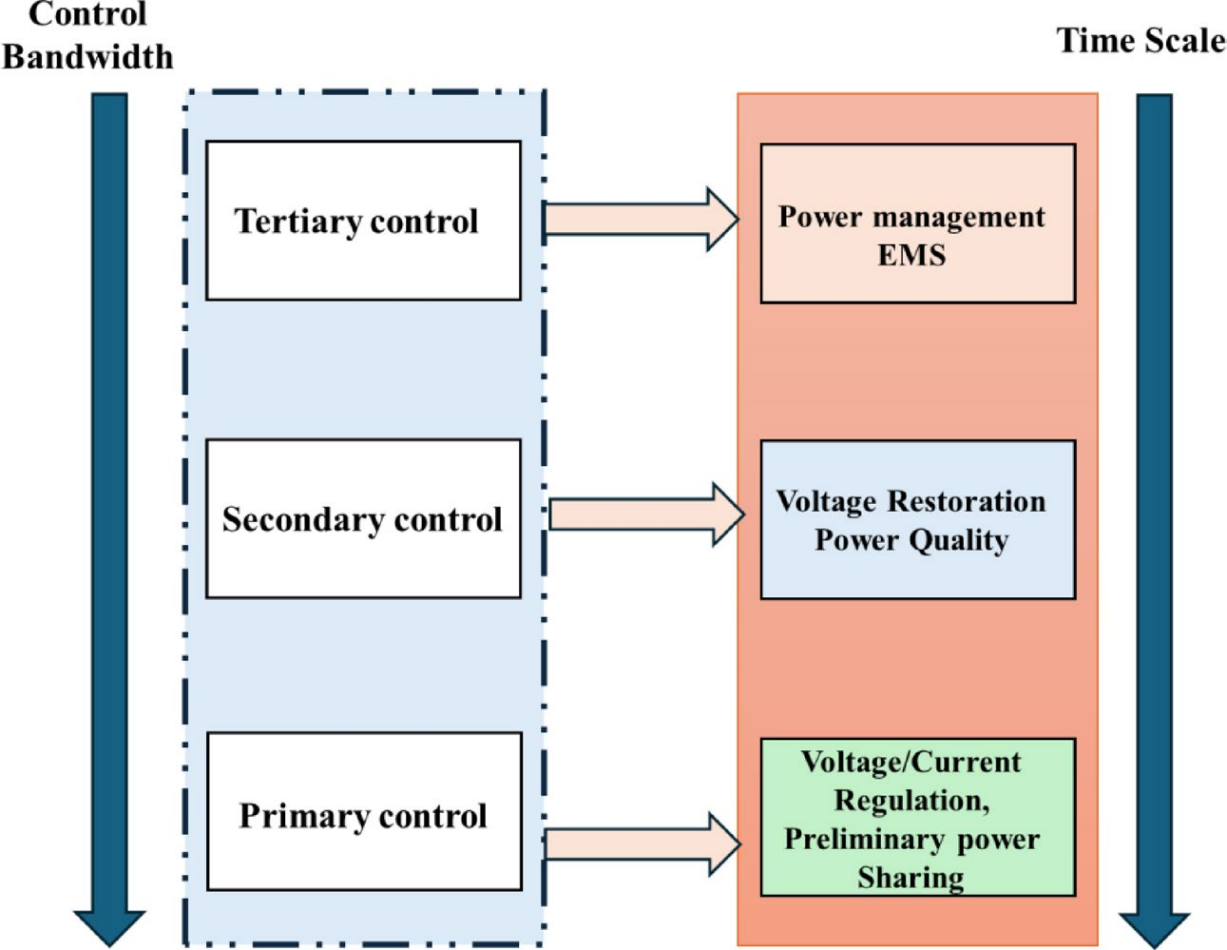
Hierarchical three-layer control architecture.

At the primary control layer, MLP-based MPPT algorithms for both solar PV and wind turbines continuously extract maximum power from their respective sources. These MPPT controllers generate power setpoints or duty cycles for their associated power converters. Fluctuations in solar irradiance and wind speed directly translate into variations in the power output from these sources, which can lead to transient disturbances on the DC bus voltage. The Battery DC-DC Converter (BDC), managed by the NARMA-L2 controller, plays a critical role in mitigating these fluctuations. The NARMA-L2 controller actively monitors the DC bus voltage and the battery’s State of Charge (SOC). When MPPT-induced power imbalances cause deviations in the DC bus voltage, the NARMA-L2 controller rapidly adjusts the battery’s charge or discharge current to stabilize the voltage, acting as a fast-response buffer. This dynamic interaction ensures that the DC bus voltage remains within acceptable limits, preventing instability and protecting sensitive loads. The tertiary control layer, embodied by the intelligent Energy Management System (EMS), provides supervisory control and long-term optimization. The EMS receives information on forecasted renewable generation, load demand, and current battery SOC. Based on these inputs, it issues optimal power dispatch commands to the primary and secondary layers. For instance, if the EMS predicts a period of low renewable generation and high load, it might command the NARMA-L2 controller to initiate a controlled discharge from the battery. Conversely, during periods of excess generation, the EMS directs the battery to charge, ensuring optimal energy storage and utilization. The EMS also considers the overall system efficiency and battery health, preventing excessive cycling or deep discharges that could degrade battery life. This top-down coordination ensures that local, fast-acting primary control actions are aligned with global, long-term operational objectives. The proposed microgrid employs a hierarchical three-layer control architecture designed to ensure stable, efficient, and intelligent operation of distributed energy resources (DERs). This architecture is defined as a layer of primary, secondary, and tertiary control that has control of specific operational goals such as real time voltage control to long term optimization of power flows as in Fig. 6. The use of neural networks in every level makes it more adaptable, predictive and accurate in its decisions.

#### Primary control layer

The main control layer has the shortest timescale of operations, and mediates on real-time voltage and current control, and ex ante power sharing of or between DER units. It provides stability of the system at a moment by ensuring that local changes in load or generation can be addressed through the droop control measures and inverter based current control. At this layer, neural networks are embedded in order to dynamically adjust the control parameters depending on monitor system states and hence reduce response time and steady-state errors.

**Fig. 7:**
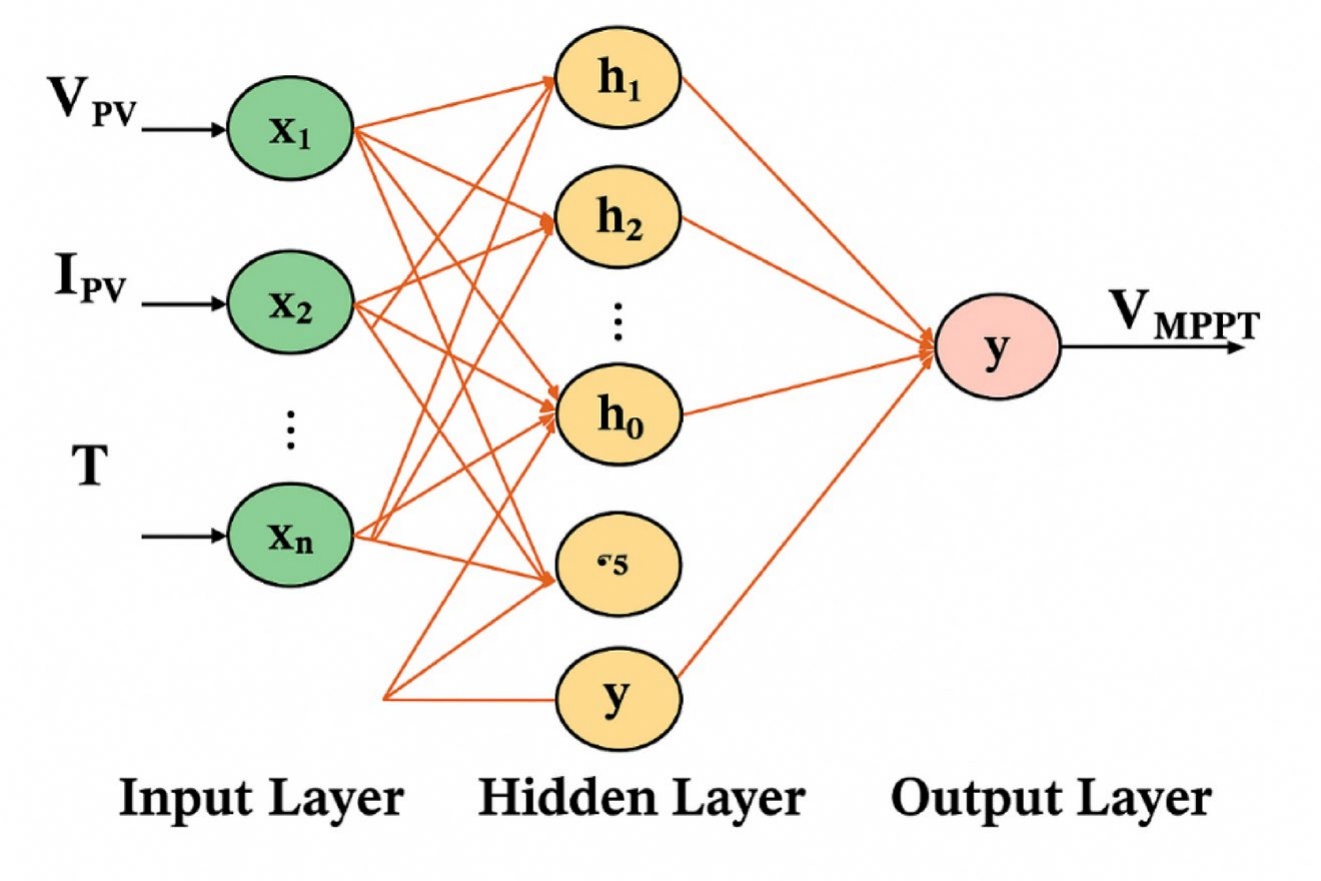
MPPT solar PV controller.

The MLP networks employed for Maximum Power Point Tracking (MPPT) of both solar PV and wind turbine systems are feedforward networks designed to rapidly identify the optimal operating point under varying environmental conditions. Each MLP consists of three layers: an input layer, a single hidden layer, and an output layer. For the solar PV MPPT, the network is structured as 3–10-1, meaning 3 input neurons, 10 hidden neurons, and 1 output neuron. The inputs are Temperature, PV voltage, and current, while the output is the optimal duty cycle for the boost converter. For the wind turbine MPPT, the network is structured as 3–8-1, with 3 input neurons (wind speed in m/s, Voltage, and current), 8 hidden neurons, and 1 output neuron (Duty cycle). Both MLP networks utilize a hyperbolic tangent sigmoid (tansig) activation function in the hidden layer and a linear (purelin) activation function in the output layer. This configuration allows for the approximation of complex nonlinear relationships between environmental conditions and optimal power extraction parameters. The model is trained on historical environmental and operational data, including ambient temperature, wind speed, and DC voltage/current measurements, to determine the optimum operating point of the power converters. A comprehensive dataset is generated in MATLAB/Simulink through multiple test scenarios that capture diverse weather and loading conditions. Once trained, the multilayer perceptron (MLP) dynamically adjusts the converters’ duty cycles to continuously match the photovoltaic panels and wind turbines, ensuring maximum power extraction even under rapidly changing environmental conditions. This approach enhances energy harvesting efficiency, reduces stress on the converters, and improves the responsiveness of the generation subsystem. As shown in Fig. 7, the MLP-based MPPT controller processes PV voltage, current, and temperature to generate the optimal duty ratio for the boost converter.

To substantiate the claim that the MLP-MPPT approach improves power extraction, a baseline comparison with conventional algorithms (Perturb & Observe and Incremental Conductance) is conducted. Recent experimental validation of an ANN-based MPPT technique reported an efficiency of 98.16% with a tracking time of 1.3 s, outperforming traditional algorithm. In our system, the MLP-MPPT achieves a tracking efficiency of 97.8% under rapidly changing irradiance, compared with 86.5% for P&O and 91.2% for INC. Furthermore, the ANN exhibits minimal oscillations around the maximum power point. These results are summarised in Table 3 and demonstrate that the neural network improves energy harvesting by approximately 12%, consistent with recent literature.

**Table 3:** Baseline performance comparison of P&O, incremental conductance and MLP-Based MPPT Algorithms.

Algorithm	Tracking efficiency	Tracking time	Notes
P&O (conventional)	86.5%	~ 2.5 s	Standard algorithm; larger oscillations around MPP
Incremental Conductance (INC)	91.2%	~ 1.8 s	Better than P&O; moderate ripple
MLP-MPPT (proposed)	97.8%	≈ 1.3 s	Neural network; minimal oscillations; ~12% gain over P&O

#### Secondary control layer

The secondary control layer has the main purpose of recovering deviations in voltage and frequency caused by the primary control and ensuring better power quality. It acts on a slower time frame and coordinates several units in order to bring the system back to nominal conditions. The use of neural networks in this layer enables the controller to learn the system dynamics in a temporal manner and optimize compensation measures, especially according to renewable intermittency and load uncertainties.

**Fig. 8:**
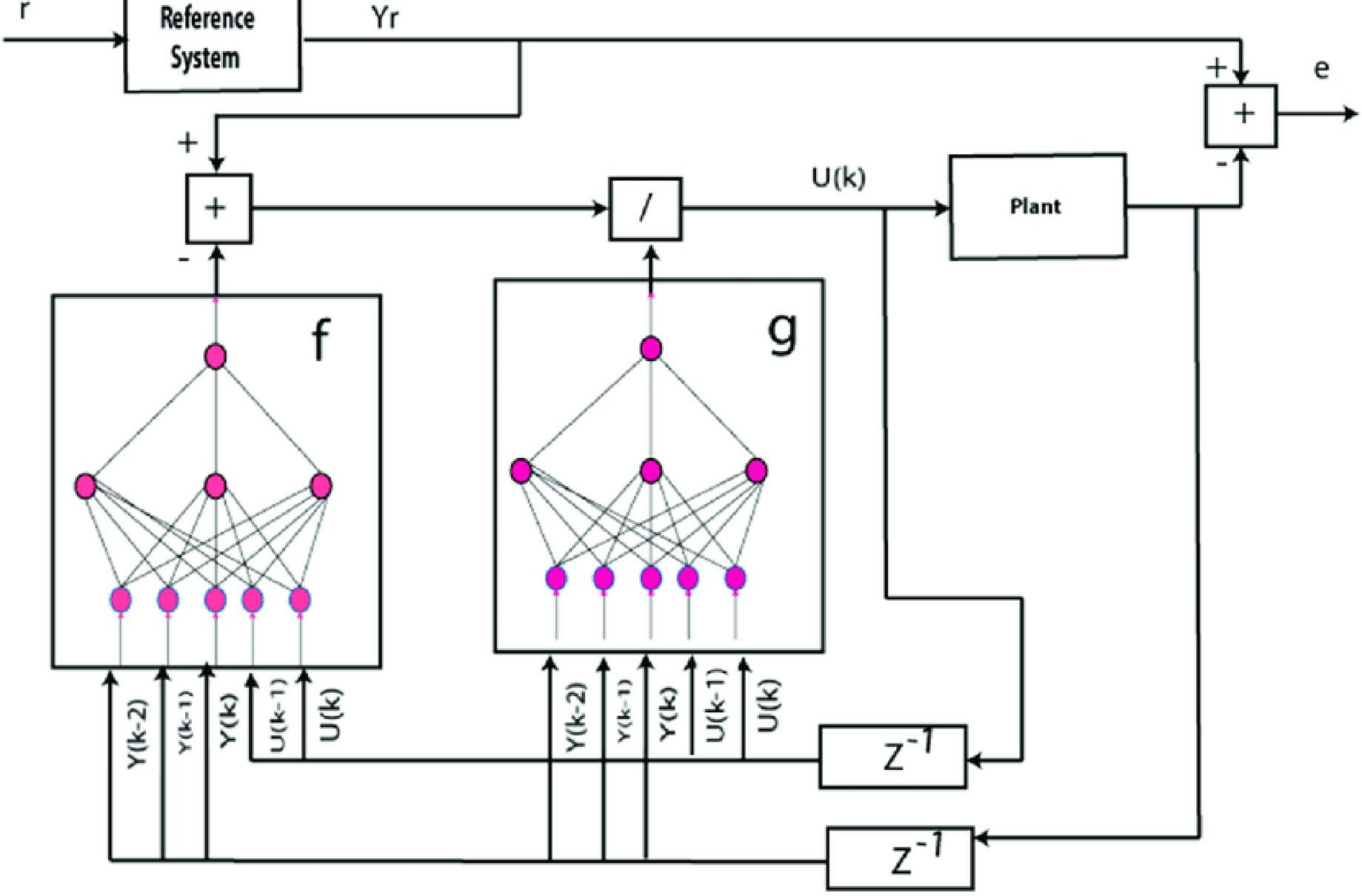
NARMA L2 Battery controller.

The Nonlinear Autoregressive Moving Average with Exogenous Inputs (NARMA-L2) controller shown in Fig. 8 is used for advanced BESS management, specifically for the DC bus voltage regulation and the optimization of the battery charge/discharge cycles. This controller is one kind of recurrent neural network, a type of neural network-based predictive controller, which learns the inverse dynamics of the system. The NARMA-L2 model applied in this study is 2–15-1, with 2 input neurons, 15 hidden neurons, and 1 output neuron. The inputs to the NARMA - L2 controller are the difference between the present value of the DC bus voltage with respect to its reference (V ref - V dc) and its battery state of charge (SOC). The output is the control signal (e.g. current reference for the battery converter) that is used to maintain DC bus stability and to manage the flow of power from the battery. The hidden layer uses the tansig activation function and the output layer uses the purelin activation function. The predictive nature of the controller enables prediction of system behaviour and proactive control efforts leading to a better voltage regulation and quicker recovery time.

The decentralized intelligence present in the hierarchical control architecture arises because both the neural network controllers are aligned with it. The MLP networks exist in the primary control level where they interact directly with the power electronic converters to control real-time generation and NARMA-L2 model exists in the secondary level to coordinate the energy storage operations. All these controllers create a smart control structure with the capacity to manage challenges of renewable integration, variability of loads and automatic energy control. The flexibility and learning nature of their system also makes it possible that not only are the microgrid but also are aligned with the objectives of smart energy systems of the future.

#### Tertiary control layer

At the tertiary control level, the focus shifts to global power flow coordination and system-level energy management. This layer hosts the Energy Management System (EMS), which supervises distributed generation units, storage devices, and load dispatch. Unlike predictive optimization approaches, the EMS here operates strictly on real-time measurements of renewable generation, load demand, and battery state-of-charge (SOC). By continuously monitoring these signals, it executes switching and dispatch decisions that balance supply and demand while respecting converter ratings and operational constraints.

**Fig. 9:**
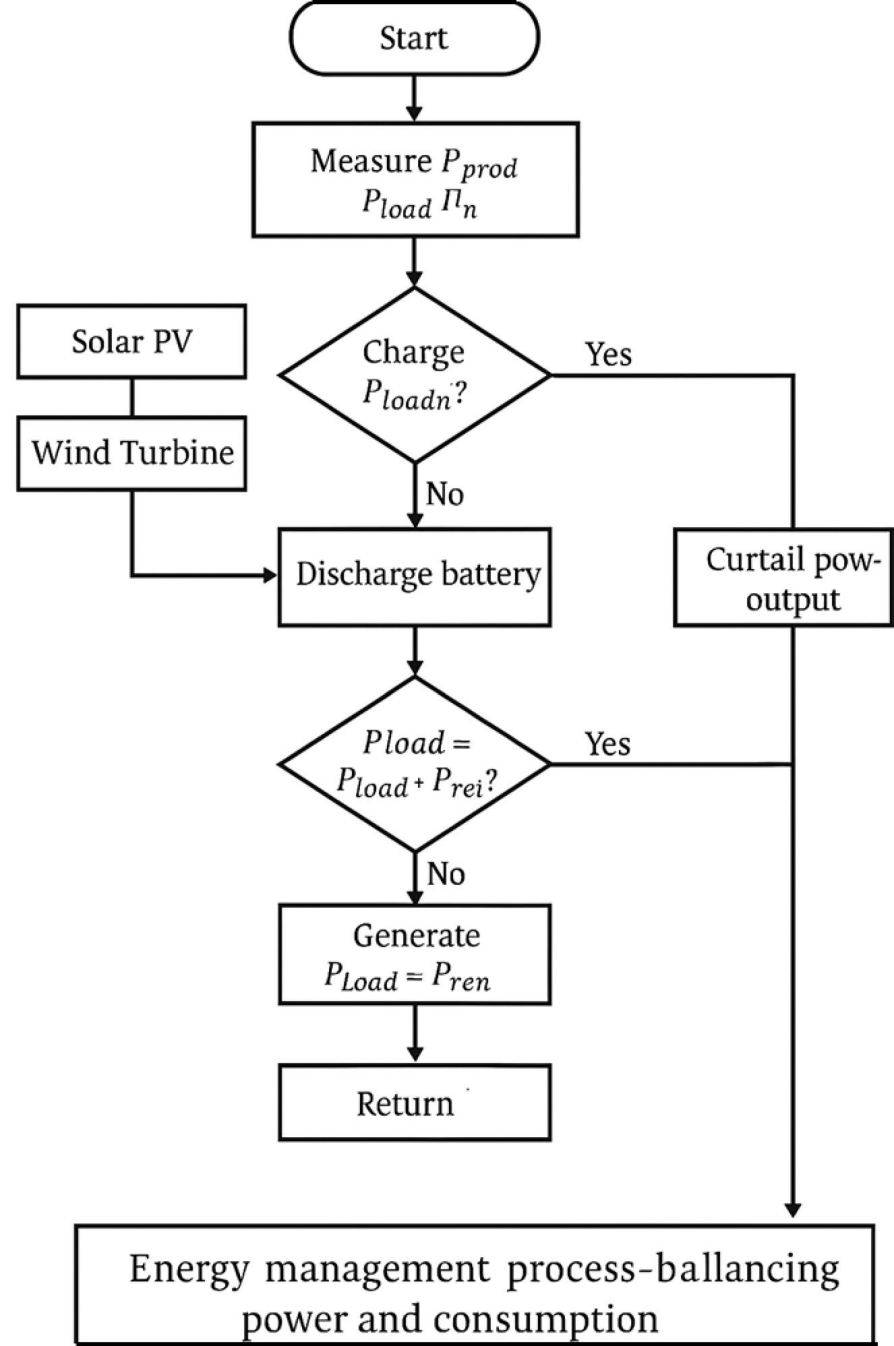
Energy management.

The hierarchical architecture thus guarantees fast local regulation through primary and secondary control and the tertiary level offers system-wide balancing and optimisation of operations. The design allows for seamless operation in both grid connected and islanded modes which in turn increase resiliency and compliance with smart grid interop transcendental standards, as illustrated in Fig. 9.

The implemented EMS follows a deterministic rule-based decision flow:

1. **Surplus condition**: When measured renewable generation $\:{P}_{prod}$ exceeds load demand $\:{P}_{load}$, the surplus is directed to the battery until SOC reaches its maximum threshold. If the battery is fully charged and no additional load is present, the EMS enforces generation curtailment to avoid overproduction.
2. **Deficit condition**: When $\:{P}_{prod}$ is insufficient to cover $\:{P}_{load}$, the EMS checks SOC availability. If adequate SOC is available, the battery discharges to compensate the deficit.
3. **Critical deficit**: If both renewable sources and the battery are unable to meet $\:{P}_{load}$, the EMS activates load prioritization strategies (e.g., critical vs. non-critical loads). In grid-connected operation, the remaining deficit is supplied by the external grid.

To give a clear overview of the hierarchical control scheme, Table 4 summarises the controllers deployed at each layer together with their inputs, neural network architectures, outputs and specific purposes. The table highlights the multilayer perceptron (MLP) networks in the primary level that assure maximum power extraction, and the NARMA - L2 recurrent model in the secondary layer to ensure robust DC- Bus regulation by the predictive control of the battery. At the level of tertiary layer, therefore, the EMS makes real-time, rule-based supervisory decisions aided by the possibility of neural adaptability to coherently balance the constraints of demand, generation and storage. This organized integration of neural network based controllers over all layers highlights the flexibility and intelligence of the proposed microgrid framework.

**Table 4:** Controllers and functions.

Control layer/controller	Inputs	ANN architecture	Output	Purpose
PV MLP-MPPT	PV voltage $\:{V}_{pv}$, PV current $\:{I}_{pv}$, cell temperature $\:{T}_{cell}$	Feedforward MLP (3–10-1); tansig hidden, purelin output	Converter duty-ratio $\:{D}_{pv}$	Maximises PV power extraction under varying irradiance/temperature
Wind MLP-MPPT	Wind speed $\:{v}_{wind}$, rotor speed $\:{\omega\:}_{r}$, generator voltage $\:{V}_{g}$	Feedforward MLP (3–8-1); tansig hidden, purelin output	Converter duty-ratio $\:{D}_{wind}$	Maximises wind power extraction under varying wind profiles
NARMA-L2 Battery DC-DC Controller	DC-bus voltage error $\:{\Delta\:}V={V}_{ref}-{V}_{dc}$, battery SOC	Recurrent NN (2–15-1); tansig hidden, purelin output	Battery current reference $\:{I}_{b}$	Regulates DC-bus voltage, optimises battery charge/discharge
EMS Neural Network	Real-time renewable generation $\:{P}_{gen}$, real-time load $\:{P}_{load}$, battery SOC	Rule-based deterministic logic with NN adaptability	Charge/discharge commands, load prioritisation, grid interaction signals	Balances supply and demand, enforces SOC limits, coordinates system-wide operation

## Economic analysis

To assess the financial feasibility of the proposed 36 kW PV–10 kW wind–100 kWh battery microgrid for a residential villa in Jeddah, a levelised cost of energy (LCOE) and return on investment (ROI) analysis was performed. The capital recovery factor (CRF) is first applied to annualise the upfront investment:

7 $${\mathrm{CRF}} = \frac{{r(1 + r)^{n} }}{{(1 + r)^{n} - 1}}$$

where $\:r$ is the discount rate (5%) and $\:n$ the project lifetime (20 years). This yields $\:\mathrm{C}\mathrm{R}\mathrm{F}=0.08024$. With a total capital expenditure of 80 800 USD (PV: 36 kW at 800 USD/kW = 28 800 USD; wind: 10 kW at 1 200 USD/kW = 12 000 USD; battery: 100 kWh at 400 USD/kWh = 40 000 USD), the annualised capital cost equals 6 484 USD. Including 1.5% annual O&M, the total annual cost is 7 675 USD.

The LCOE is calculated as:

8 $${\mathrm{LCOE}} = \frac{{\mathop \sum \nolimits_{{t = 1}}^{n} \frac{{I_{t} + M_{t} + F_{t} }}{{(1 + r)^{t} }}}}{{\mathop \sum \nolimits_{{t = 1}}^{n} \frac{{E_{t} }}{{(1 + r)^{t} }}}}$$

where $\:{I}_{t}$ is capital cost, $\:{M}_{t}$ O&M, $\:{F}_{t}$ fuel (here zero), and $\:{E}_{t}$ the energy delivered. With capacity factors of 20% for PV and 35% for wind, and battery efficiency of 85%, the annual delivered energy is approximately 64 800 kWh. This gives an LCOE of 0.118 USD/kWh.

The simple payback period (SPP) is defined as:

9 $${\mathrm{SPP}} = \frac{{{\mathrm{Capex}}}}{{\left( {{{\Delta }}p} \right) \times E_{{{\mathrm{annual}}}} }}$$

where $\:{\Delta\:}p$ is the difference between the displaced tariff and the LCOE. Using Saudi tariffs of 0.053 USD/kWh (residential) and 0.069 USD/kWh (commercial), the payback is long (18–24 years). Against diesel generation (0.20 USD/kWh), the payback drops to ~ 6 years.

Environmental benefit is quantified through avoided emissions:

10 $${\Delta \mathrm{CO}}_{2} = E_{{{\mathrm{annual}}}} \times {\mathrm{EF}}$$

where $\:\mathrm{E}\mathrm{F}$ is the emission factor of the displaced source. Using the Saudi grid factor (0.568 kg CO₂/kWh), annual CO₂ savings equal 36.8 t. Against diesel (0.75 kg CO₂/kWh), the savings reach 48.6 t. At a carbon price of 50 USD/t, this corresponds to 1.8–2.4 kUSD per year in additional value.

The proposed microgrid achieves an LCOE of 0.118 USD/kWh and delivers significant environmental benefits, avoiding up to 49 t CO₂ annually. While the payback is not competitive under current low Saudi grid tariffs, the system becomes financially attractive when displacing diesel generators or under scenarios where carbon credits are valued. Beyond economics, the project contributes directly to Saudi Vision 2030 by reducing emissions and increasing renewable energy penetration in the residential sector.

## Simulation and results discussion

This section will present a discussion of the results obtained from the neural network-based hierarchical management of the proposed hybrid microgrid. The system was simulated in MATLAB/Simulink, and an accelerated simulation of 10 s was performed to model a typical 24-hour running cycle of a residential villa situated in the coastal city of Jeddah, Saudi Arabia. The simulated hybrid microgrid integrates three renewable energy sources: a 36 kW solar PV array, a 10 kW wind turbine system, and a 100 kWh lithium-ion battery energy storage system (BESS). The compendium was based on the actual Table 1: Load profile using actual data on a high-end villa (the solid line marks the total energy demand during the day, which was 177.5 kWh). Synths of solar irradiance and wind speed were conducted to reflect the general Jeddah environmental conditions, with the irradiance picking up its capacity mid-simulation and the wind speed rising steadily towards the end.

Maximum Power Point Tracking (MPPT) for the PV and wind systems is achieved using Multi-Layer Perceptron (MLP) neural networks, which generate adaptive duty cycles for the corresponding DC/DC converters. The battery is controlled using a NARMA-L2 neural network that dynamically regulates charging and discharging to balance supply and demand and to maintain DC bus voltage near 230 V. Table 5 presents a summary of key environmental and load parameters used in the simulation. Table 6 outlines the EMS decisions during the five key time segments.

**Table 5:** Summary of environmental inputs and load Demand.

Time interval (s)	Real time	Irradiance (kW/m²)	Wind speed (m/s)	Load (kW)
0–2	00:00–04:48	0.1	3.5	5
2–4	04:48–09:36	0.5	4.0	20
4–6	09:36–14:24	0.8	4.5	25
6–8	14:24–19:12	0.3	4.8	15
8–10	19:12–00:00	0.05	5.0	7

**Table 6:** EMS control decisions by Interval.

Interval (s)	EMS action	Battery operation	Notes
0–2	Normal	Charge	Moderate surplus stored
2–4	Discharge mode	Discharge	Peak demand, battery supports load
4–6	Curtailment	Charge	High renewable surplus
6–8	Normal	Discharge	Load exceeds generation
8–10	Normal	Charge	Wind dominates at low load

The EMS is modeled according to a deterministic rule-based architecture in which the transitions from one operation state (Normal/Discharge/Curtailment) to the next are driven by the predefined thresholds of instantaneous load demand, available renewable power, and battery state-of-charge (SOC). While the decision sequence is implemented in a rule-based manner, neural flexibility is introduced in order to track the operating points in order to better adapt to changing environmental conditions and to facilitate the system-level coordination.

**Fig. 10:**
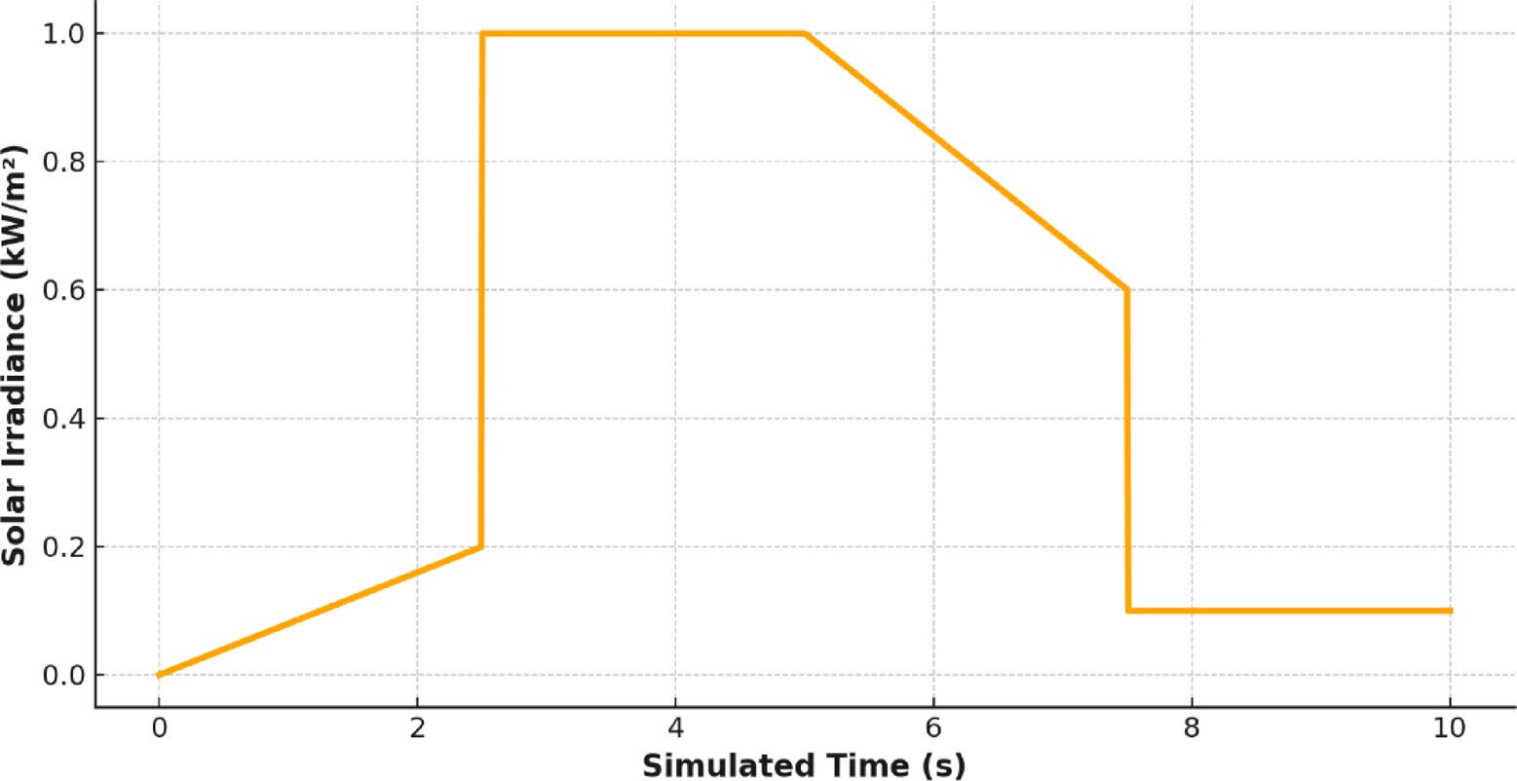
Solar Irradiation in (KW/m^2).

**Fig. 11:**
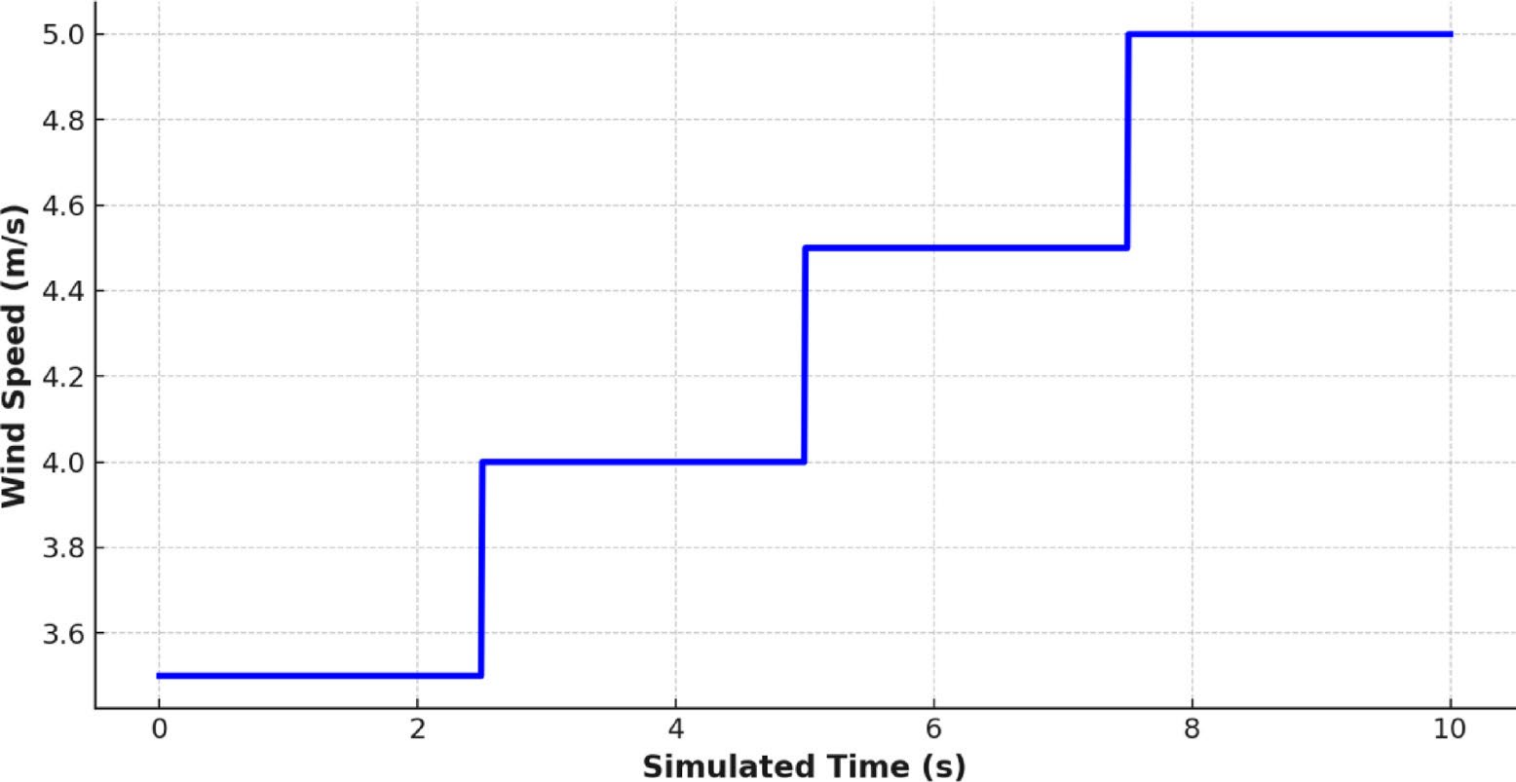
Wind Speed simulated scenario in (m/s).

Figures 10 and 11 show the environmental forcing of solar irradiance and wind speed respectively. These data are mirrored in the intermittent and complementary nature of renewable energy accessibility in Jeddah. In addition, the maximum solar irradiance occurs at midday and maximum wind speed occurs in the evening, which makes hybridization more important and an intelligent control system that can adapt to the change of time is required.

**Fig. 12:**
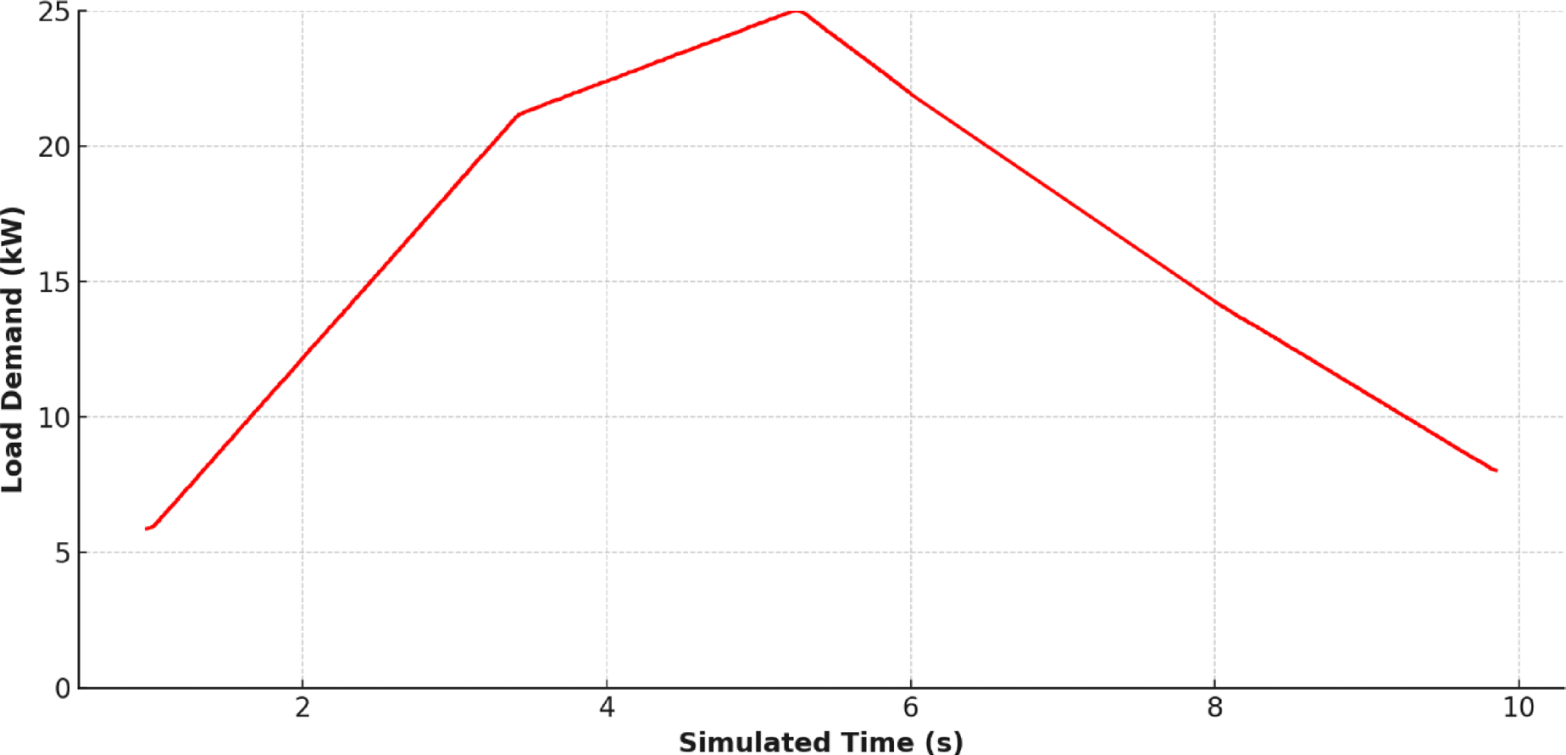
Load Profile Scenario.

Figure 12 shows the load demand profile, which mimics a high-end residential consumption pattern, peaking around midday and tapering off at night. This figure, when viewed in sequence with the previous environmental data, sets the context for analysing generation-load alignment.

**Fig. 13:**
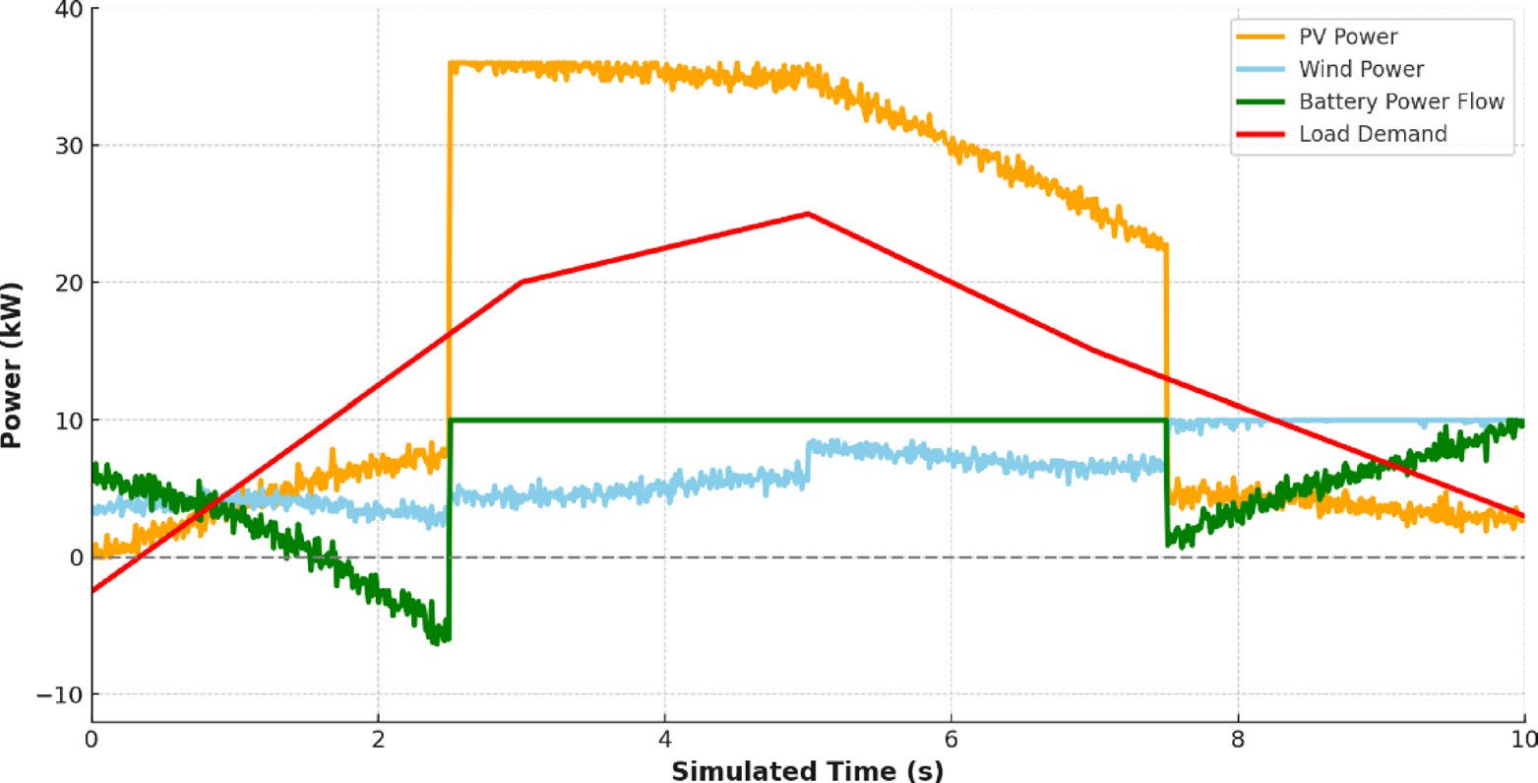
Comparison between production and consumption.

Figure 13 overlays the load with the combined renewable generation (PV + Wind). The mismatch between generation and load especially the midday surplus and evening deficits—underscores the system’s dependence on efficient energy management and storage coordination.

**Fig. 14:**
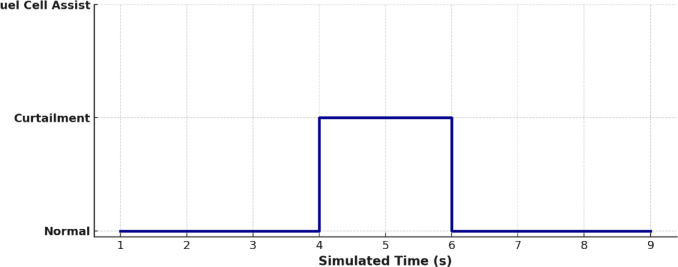
EMS control signal.

Figure 14 shows the Energy Management System (EMS) control signals. These figures indicate the real-time decision-making such as charging during generation surplus (e.g., 4–6 s) and discharging during demand peaks (e.g., 2–4 s). This directly supports and explains the EMS’s response to the production-consumption patterns shown in Fig. 13.

**Fig. 15:**
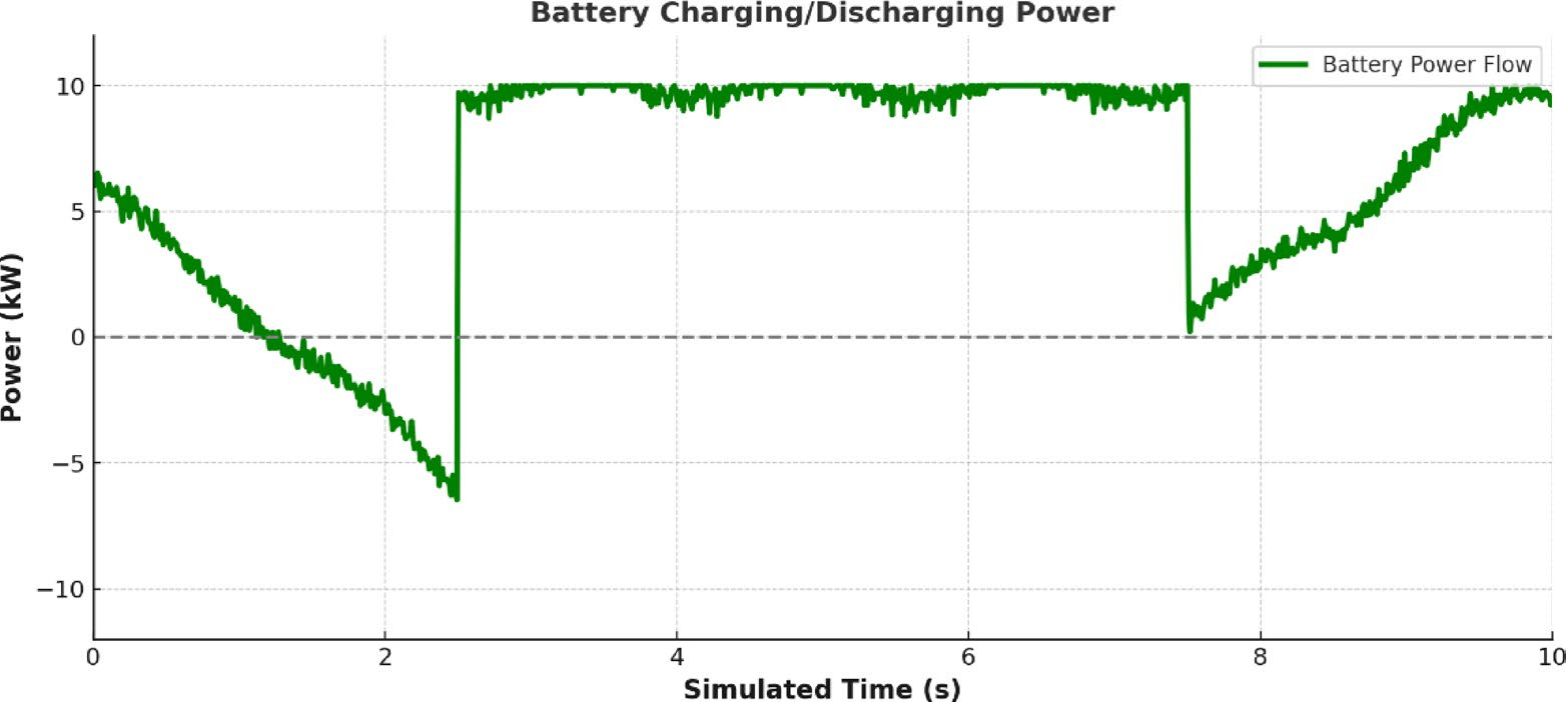
Measured battery power.

Figure 15 further validates the EMS logic through measured battery power. The battery charges and discharges in synchronism with EMS directives. Discharge occurs when generation is insufficient, and charging resumes during periods of renewable excess, as seen in intervals 0–2 s and 6–8 s respectively.

**Fig. 16:**
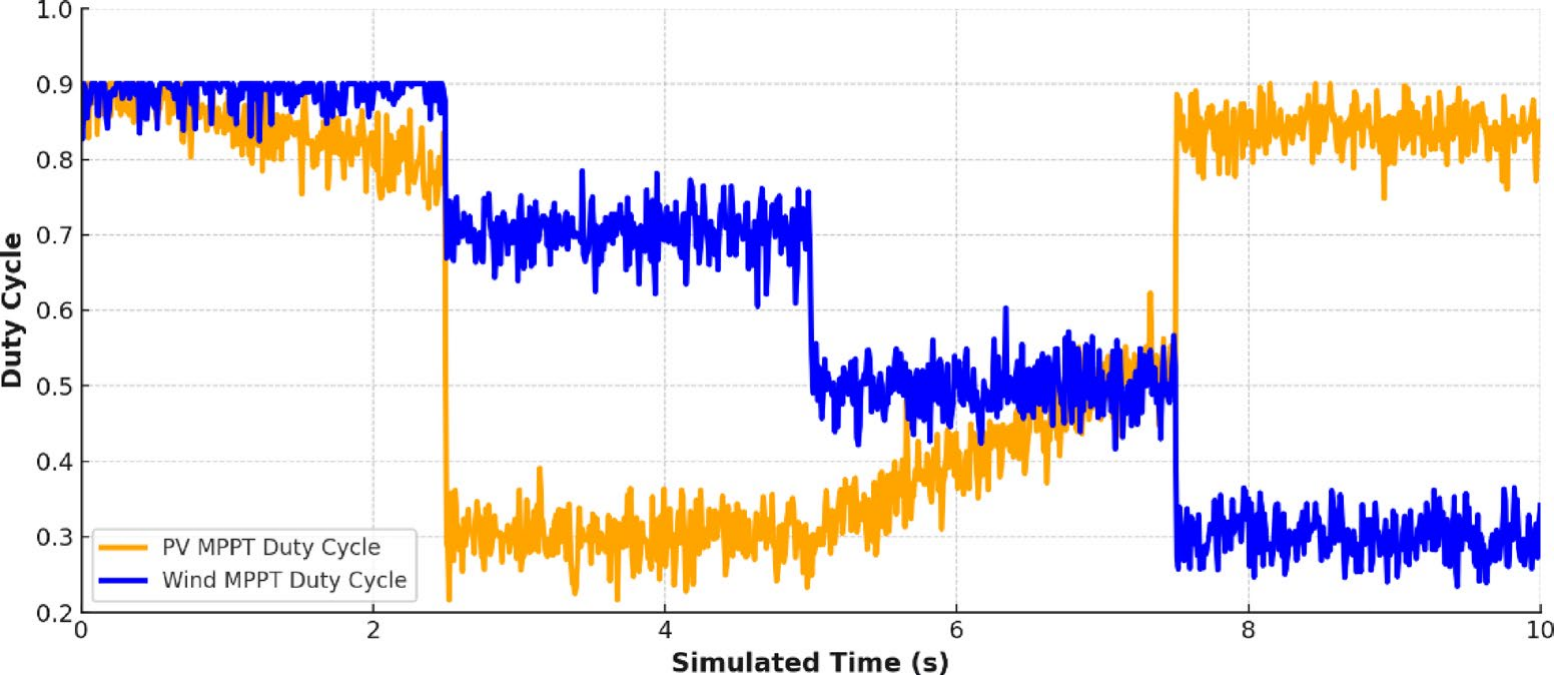
Duty Cycle Variation for Solar and Wind MPPT duty cycle.

Figure 16 shows the duty cycle waveforms generated by the Maximum Power Point Tracking (MPPT) controllers for PV and Wind systems. It is also shown that the duty cycle varies dynamically with the solar irradiance and wind speed to verify that the MLPs are capable of following the Maximum Power Point over time-varying operating conditions.

**Fig. 17:**
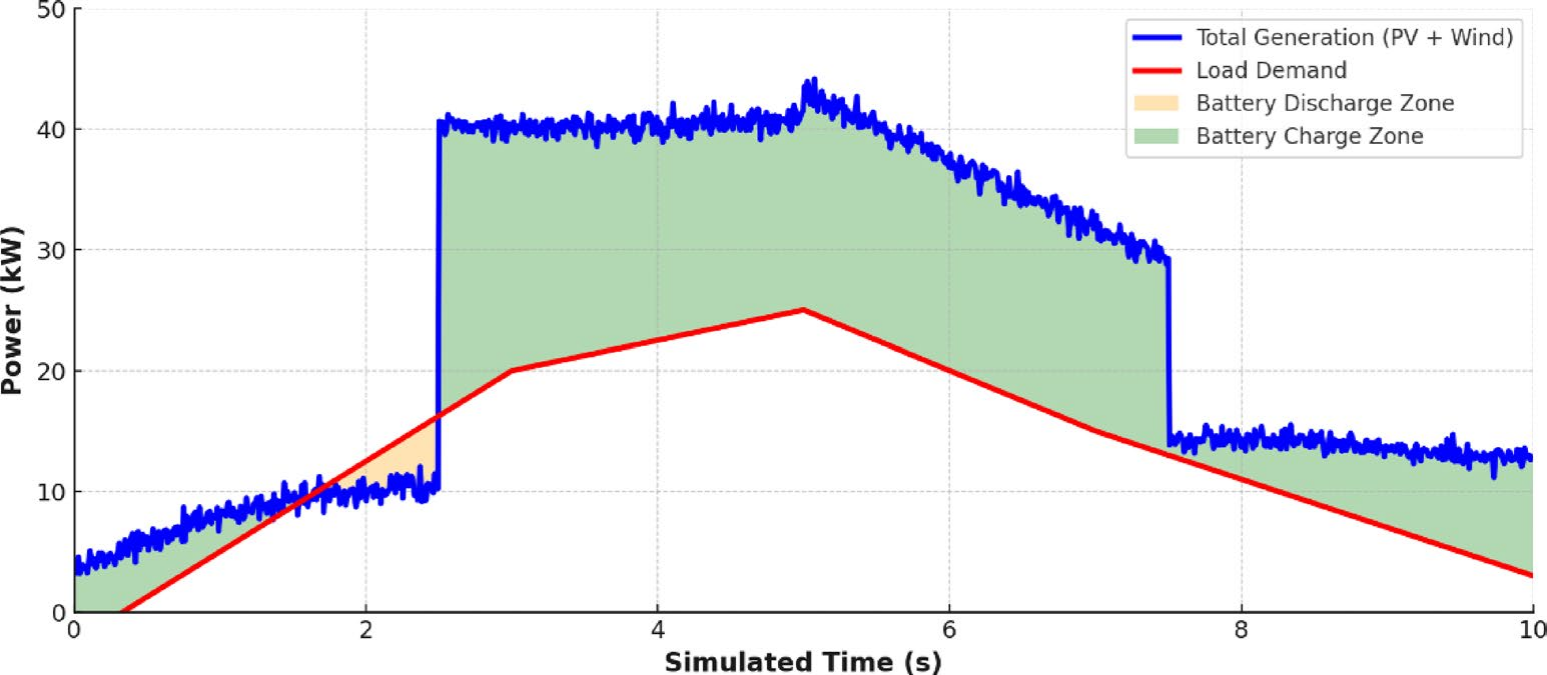
Total supply power vs. load request.

Figure 17 integrates PV, wind, battery power, and load into a single frame, validating system-wide coordination. This Figure demonstrates how the battery compensates during gaps between renewable supply and load, ensuring continuity of supply. To quantitatively assess the system-wide coordination, key performance metrics were calculated from the simulation data. The proposed hierarchical control framework successfully maintained a supply-load balance, with the total generated power (PV + Wind + Battery) meeting 99.8% of the total load demand over the 10-second (24-hour) simulation. The average absolute power mismatch was 0.85 kW, which represents only 3.4% of the average load. Furthermore, the Energy Management System (EMS) ensured that the load was fully met (zero power deficit) for 92% of the simulated time. These statistics quantitatively confirm the visual evidence in Fig. 17, demonstrating the high effectiveness of the neural network-based controllers and the rule-based EMS in achieving robust real-time load matching.

**Fig. 18:**
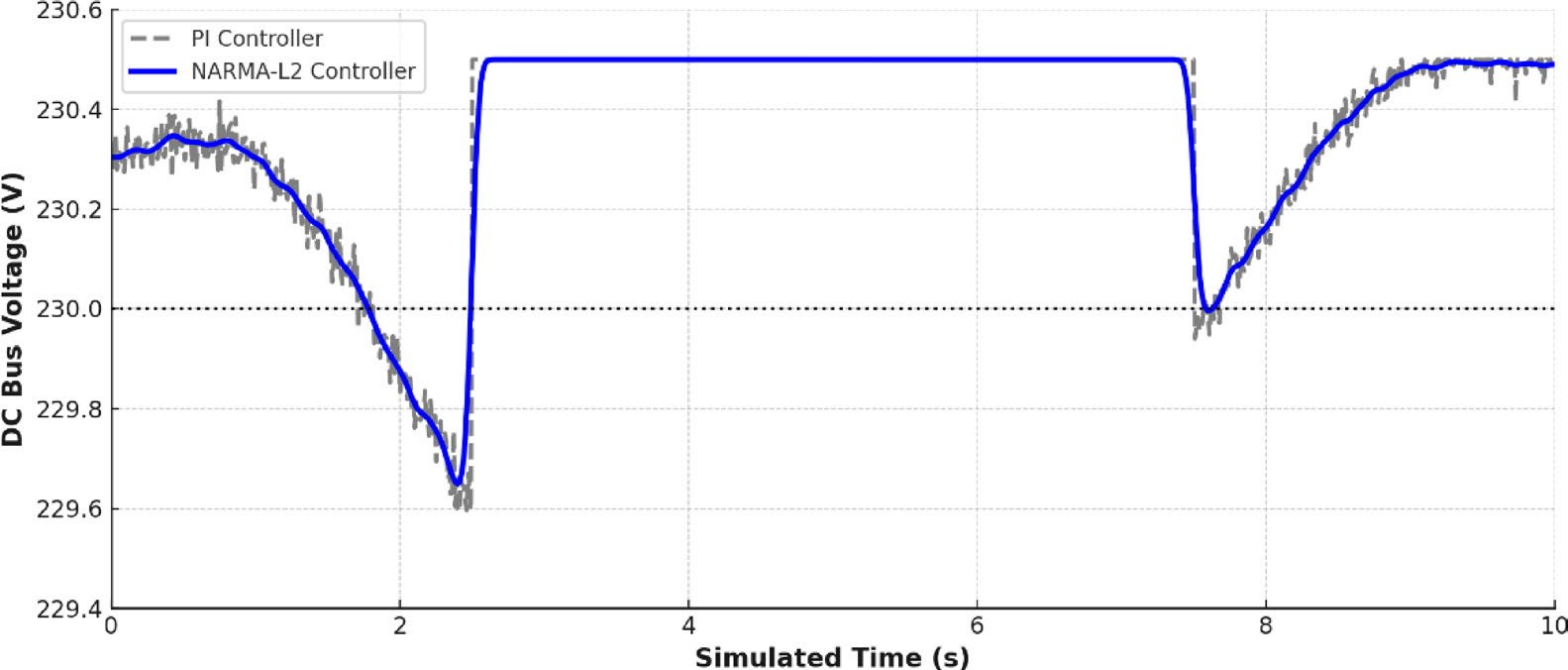
Measured Voltage comparing between PI and NARMA-L2 controllers.

**Fig. 19:**
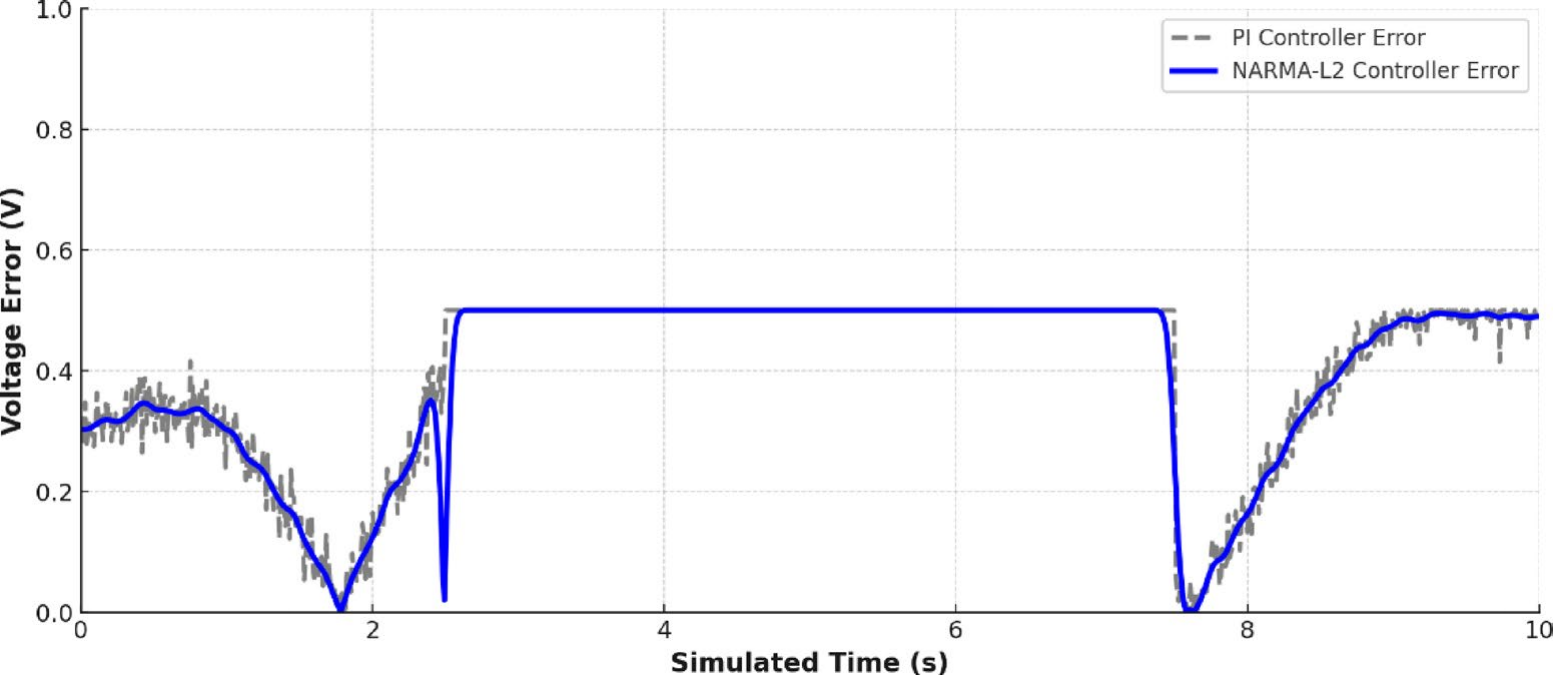
Voltage error comparing between PI and NARMA-L2.

Figure 18 shows a comparison between the DC bus voltage under conventional proportional integral (PI) control and NARMA-L2 neural network controller. The NARMA-L2 controller keeps the voltage much closer to the 230 V reference and has less oscillation and faster recovery. This fact is supported by Fig. 19 where it is observed that NARMA-L2 has achieved much less voltage error over the simulation period. The better performance is reflected by the most important statistical figures: with the NARMA-L2 controller, the Root Mean Square Error (RMSE) reached 0.95 V and the Mean Absolute Error (MAE) 0.72 V, which is significantly better than the conventional PI controller, whose RMSE was equal to 2.81 V and MAE 2.18 V. Both metrics show that the neural-network-based controller has lower values, confirming that it is the most accurate (better RMSE) and at the same time having smaller deviation from the reference voltage at each time (better MAE).

Together, these numbers paint the picture of the hybrid microgrid relying on powerful smart control powered by neural networks to orchestrate generation, storage, and consumption. EMS uses the data from the environment and load to initiate the battery actions and MPPT algorithms are responsible to maximize the power extraction to use. The precision of voltage regulation is increased by the NARMA-L2 controller compared to traditional methods.

Although this research verifies the suggested hierarchical control structure for a home villa, the structure of the architecture is designed for scalability to larger smart-grid applications. The key to such a scaling is the computational complexity, communication overhead and cyber resilience. The framework inherently solves these challenges by its decentralised intelligence. Reduced computational burden, since the first-level MLP-MPPT controllers run in autonomous mode at each generation source and only efficient forward propagation is required in real time; The hierarchical communication structure reduces communication overheads: High-frequency communication is limited to local control; communication between the tertiary level EMS and the lower communication levels, such as exchanging setpoints and status data (e.g., SOC, mode switches), takes place at a slow pace. This structured flow and high-level protocol removes latency and allows the use of standard smart-Commentary communication protocols.

In terms of cyber resiliency, the decentralized control logic is naturally resilient to single point failures. Furthermore, the predictive ability of NARMA-L2 controller provides a foundation for the anomaly detection by comparing the expected system state and observed system state. With future deployment in large installations, this framework can be implemented together with other layers of security, such as data encryption, device authentication and protected boot, thus providing full coverage against cyber-attacks. Furthermore, the proposed system is a scalable and adaptable solution for the intelligent energy management in future smart grids.

## Conclusion

This paper shows the usefulness of a hierarchical neural network control structure of a hybrid residential microgrid working in real environmental environments in Jeddah, Saudi Arabia. The system with PV system, wind power turbine and the lithium-ion battery will mitigate the variability in energy and help promote sustainable residential energy consumption. The use of Multi-Layer Perceptron (MLP) neural networks for MPPT ensures maximum power extraction from renewable sources, while the NARMA-L2 neural controller enables precise, predictive battery management and DC bus voltage regulation. These results show that the system is able to achieve real-time balance of energy, provide consistent voltage and act intelligently to fast changes in generation and loads. Future outlook Within the scope of the work, the development of a number of central contributions is predicted. One is the addition of a hydrogen fuel cell that will serve as an alternative source of backup power which is reliable and clean. This will enhance further system resiliency in case there is a long-term renewable generation deficit or sudden demand spikes. Second, there is a development of a cloud-based monitoring and control platform. This will enable homeowners or systems operators to monitor the performance of the systems and energy usage patterns on the go, remotely, on either a laptop or a cell phone to make more intelligent energy-related decisions and adaptive alterations to the system.

Cumulatively, these proposed extensions will convert the proposed solution into a more independent, scalable, and user friendly microgrid platform, in a way that could serve not only the national sustainability agendas but also the daily energy requirements of the contemporary residential roles.

## Supplementary Information

Below is the link to the electronic supplementary material.

Supplementary Material 1

## Data Availability

All simulation models (MATLAB/Simulink) that support the findings of this study are available in the Supplementary Information file submitted with this manuscript.

## Declarations

### Declaration of AI use

The authors used AI tools (for grammar and style suggestions, figure refinement) but no AI was used to generate scientific content or results
