# Supplementary figures and images for: Artificial neural network based hierarchical intelligent control framework for a residential microgrid
Source: Sci Rep. 2025 Nov 23;15:45174. doi: 10.1038/s41598-025-29034-x (PMC12749602; doi:10.1038/s41598-025-29034-x)

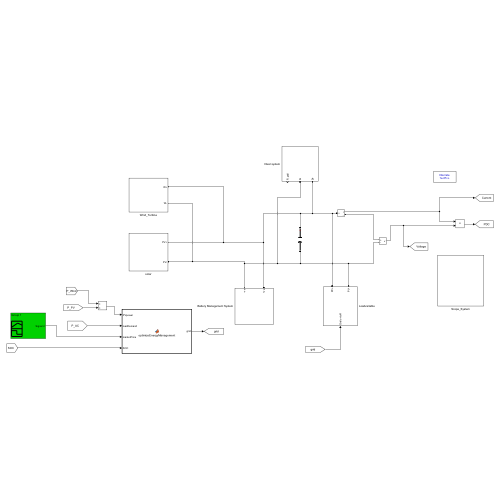

Supplement: Supplementary file 1 — Supplementary Material 1 [file 41598_2025_29034_MOESM1_ESM.slx › metadata/thumbnail.png]
